# Multiomics analyses reveal that PRMT5 regulates membrane transport and cholesterol synthesis in white adipocytes

**DOI:** 10.1002/imo2.70055

**Published:** 2025-10-05

**Authors:** Xiyue Chen, Zhihao Jia, Xiashiyao Zhang, Feng Yue, James F. Markworth, Christina R. Ferreira, Jun Wan, Shihuan Kuang

**Affiliations:** ^1^ Department of Animal Sciences Purdue University West Lafayette Indiana USA; ^2^ Cambridge‐Suda Genomic Resource Center, Suzhou Medical College Soochow University Suzhou China; ^3^ Department of BioHealth Informatics, Luddy School of Informatics and Computing Indiana University at Indianapolis Indianapolis Indiana USA; ^4^ Metabolite Profiling Facility, Bindley Bioscience Center Purdue University West Lafayette Indiana USA; ^5^ Department of Medical and Molecular Genetics Indiana University School of Medicine Indianapolis Indiana USA; ^6^ Center for Computational Biology and Bioinformatics Indiana University School of Medicine Indianapolis Indiana USA; ^7^ Department of Orthopedic Surgery and Department of Pathology Duke University School of Medicine Durham North Carolina USA; ^8^ Duke Cancer Institute Durham North Carolina USA

**Keywords:** adipocytes, cholesterol synthesis, membrane transport, protein methylation

## Abstract

The adipose tissue (AT) is a main regulator of systemic energy homeostasis, and AT ﻿dysfunction leads to insulin resistance and other metabolic complications. Protein arginine methyltransferase 5 (PRMT5) catalyzes symmetrical dimethylation of arginine residues to modulate protein stability and function. Besides its well‐studied oncogenic functions, PRMT5 has recently been shown to play a physiological role in AT through poorly understood mechanisms. Here, we explore the function of PRMT5 in AT through unbiased RNA sequencing and lipidomic analyses of AT in wild‐type and adipocyte‐specific *Prmt5* knockout (*Prmt5* ^*AKO*^) mice. Transcriptomic profiling revealed that *Prmt5* ^*AKO*^ alters the expression of genes related to metabolism and membrane transport. Specifically, *Prmt5* ^*AKO*^ induces genes enriched in glucose transport and glycolysis pathways, while suppressing genes encoding fatty acid (FA) transporters. Lipidomics analysis showed altered compositions of triacylglycerols (TAGs), fatty acids (FAs), and glycerophospholipids. Additionally, *Prmt5* ^*AKO*^ promotes cholesterol biogenesis, associated with hyperlipidemia and hepatic steatosis in mice. These multiomics approaches uncover previously unappreciated roles of PRMT5 as an epigenetic regulator of metabolic homeostasis via coordinating membrane transport, balancing glucose and FA metabolism, and promoting cholesterol biosynthesis. This study highlights a novel mechanism by which protein methylation regulates systemic energy balance.

## Highlights

- *Prmt5* ^*AKO*^ downregulates genes related to fatty acid (FA) uptake, triacylglycerol (TAG) biosynthesis, and FA oxidation, while upregulating genes involved in glucose transport and glycolysis.
- In addition, *Prmt5* ^*AKO*^ upregulates genes involved in peroxisomes and cholesterol biogenesis.
- These changes led to alterations in lipid composition, notably reduced TAG content and selective increases in glycerophospholipid and sphingolipid species.

## Graphical abstract

*Prmt5* ^*AKO*^ downregulates genes related to fatty acid transport and triacylglycerol synthesis, while upregulating genes involved in glucose transport and cholesterol biogenesis. These changes cause alternation in lipid composition and metabolic phenotypes of *Prmt5* ^*AKO*^ mice.

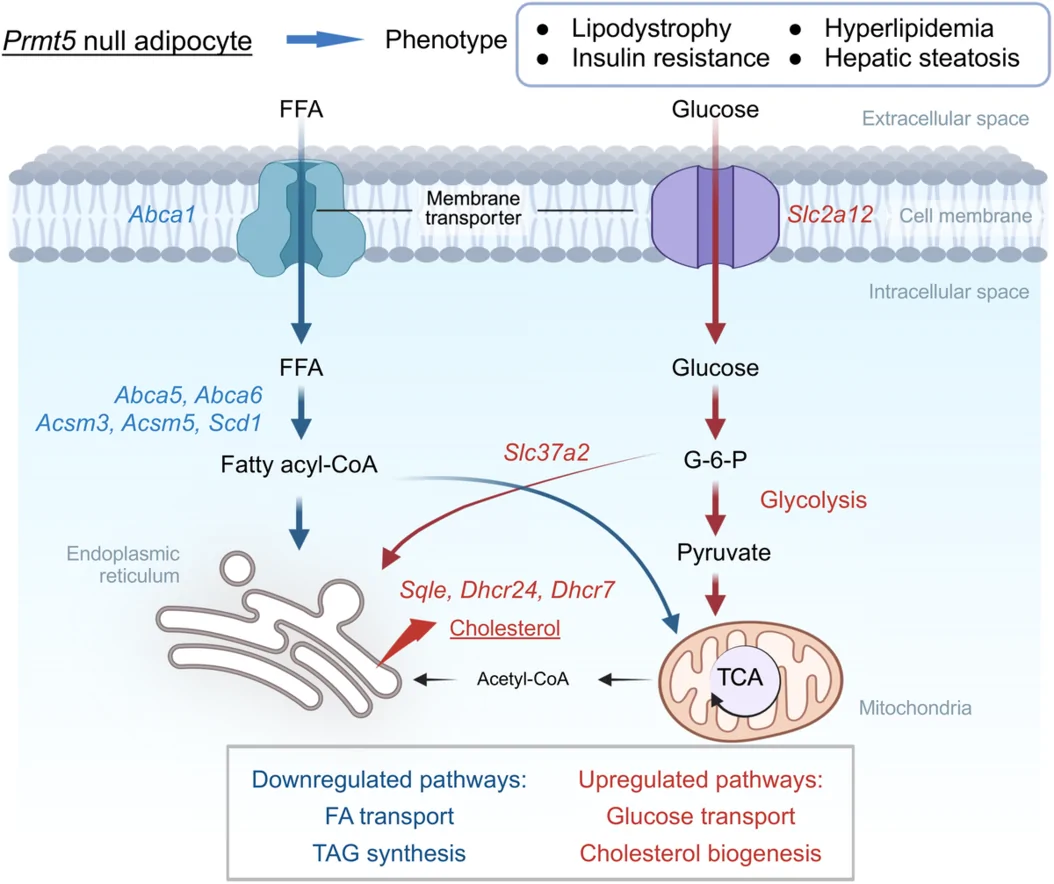

## 1 INTRODUCTION

In recent decades, the global incidence of obesity has increased dramatically due to overnutrition and sedentary lifestyles, increasing risks of metabolic disorders such as cardiovascular disease and type 2 diabetes (T2D) [1]. Adipose tissue (AT) plays a critical role in systemic energy homeostasis by dynamically remodeling in response to metabolic cues to regulate lipid storage, mobilization, and signaling transduction [2]. In humans and rodents, there are three types of ATs: white adipose tissue (WAT), brown adipose tissue (BAT), and beige adipose tissue (BeAT). WAT primarily functions as an energy reservoir, storing excess nutrients in the form of triacylglycerols (TAGs) within lipid droplets (LDs) and releasing free fatty acids (FFAs) through lipolysis during energy deficit [3]. In contrast, BAT is specialized for energy expenditure through non‐shivering thermogenesis. It is rich in mitochondria and expresses uncoupling protein 1 (UCP1), which dissipates energy as heat [4]. BeAT arises from WAT upon cold stimulation and shares thermogenic features with BAT. While BAT and BeAT contribute to adaptive thermogenesis and metabolic flexibility, WAT comprises the majority of AT mass in adult humans [5]. Importantly, WAT also functions as an endocrine organ by secreting hormones and cytokines, including leptin, adiponectin, and lipid‐derived signaling molecules, to regulate systemic energy metabolism and inter‐organ communication.

Adipocytes dynamically regulate cellular lipid dynamics through coordinated lipid uptake, synthesis, breakdown, and release. Circulating FFAs are taken by adipocytes to esterify into TAGs and stored in LDs. These LDs are comprised of a core of neutral lipids, primarily TAGs, but also ceramides, cholesteryl esters (CEs) and other lipid species. LDs are surrounded by a glycerophospholipid monolayer that differs from other bilayer membranes, such as those of endoplasmic reticulum (ER) membrane [6]. When energy demand increases, TAGs are hydrolyzed into fatty acids (FAs) and glycerol, which are released into the bloodstream and utilized by peripheral tissues. Thus, LD‐mediated lipid dynamics are essential for cellular homeostasis and worth investigation.

Cellular FA transport is mediated by passive diffusion or protein‐mediated mechanisms [7]. Upon stimulation, FA transporters, such as CD36 and fatty acid transport protein (FATPs), translocate to the plasma membrane to enhance FA uptake, a mechanism essential for postprandial lipid clearance and rapid energy supply to peripheral tissues [7, 8, 9]. CD36 is highly expressed in AT and is genetically linked to hyperlipidemia and hypertension [8]. ﻿FATP1 and FATP4 also facilitate FA uptake by coupling it with acyl‐CoA synthesis [9]. Dysregulation of these transporters contributes to metabolic diseases like T2D, highlighting them as potential therapeutic targets [7].

In addition to TAGs, adipocytes also synthesize and release other bioactive lipids, including glycerophospholipids and cholesterols derivatives. These molecules contribute to membrane architecture, intracellular signaling, and lipid trafficking. Among the glycerophospholipids, phosphatidylcholine (PC) is the most abundant component of the LD membrane [10, 11], followed by phosphatidylethanolamine (PE), phosphatidylinositol (PI), and phosphatidylserine (PS) [12]. The biosynthesis, interconversion, and compartmentalization of these phospholipids are closely linked to organelle function and LD dynamics [13, 14]. As such, understanding the regulation of lipid composition and membrane transport is critical to uncover the molecular bases of AT function in health and disease.

Protein arginine methyltransferases (PRMTs) catalyze protein arginine methylation, as key enzymes of posttranslational modifications (PTMs). PRMTs regulate many cellular activities, including RNA processing, DNA repair, and protein–protein interactions [15]. Based on their methylation preferences, PRMTs are classified into three types: type I enzymes produce asymmetric dimethylarginine, type II enzymes like PRMT5 generate symmetric dimethylarginine, and type III enzymes catalyze monomethyl arginine [16]. PRMT5, as the major type II enzyme, modifies both histone and non‐histone proteins, thereby playing critical roles in epigenetic regulation and signal transduction [17]. PRMT5 is best known for its oncogenic functions, with high expression in multiple tumor types. Accordingly, several PRMT5 inhibitors are currently in clinical trials as anticancer therapeutics [18]. More recently, PRMT5 has emerged as a critical regulator of metabolic homeostasis. In cardiomyocytes, PRMT5 maintains cardiac function by repressing protein O‐GlcNacylation [19]. And in hepatocytes, it promotes gluconeogenesis through CREB methylation [20]. Moreover, recent studies suggest that PRMT5 is associated with metabolic diseases like T2D, where it modulates insulin signaling and pancreatic β‐cell function [21].

In adipose tissue, PRMT5 has been shown to regulate adipogenic and lipogenic gene expression via PPARγ‐dependent mechanisms in vitro [22]. Our lab previously reported that adipocyte‐specific *Prmt5* knockout (*Prmt5* ^AKO^) impairs LD formation and TAG synthesis in WAT [23]. However, the mechanisms by which PRMT5 regulates lipid composition and membrane transport in vivo remain poorly understood.

In this study, we aim to investigate the physiological role of PRMT5 in white adipocytes (WAs) by integrating transcriptomic and lipidomic profiling in *Prmt5* ^AKO^ mice. We specifically hypothesize that PRMT5 modulates key membrane transport pathways and lipid homeostasis. Our findings reveal that PRMT5 acts as a critical epigenetic regulator of membrane transport and lipid handling, thereby linking protein methylation to systemic metabolic homeostasis.

## 2 RESULTS

### Transcriptional changes in response to *Prmt5* KO in WAs

**Figure 1:**
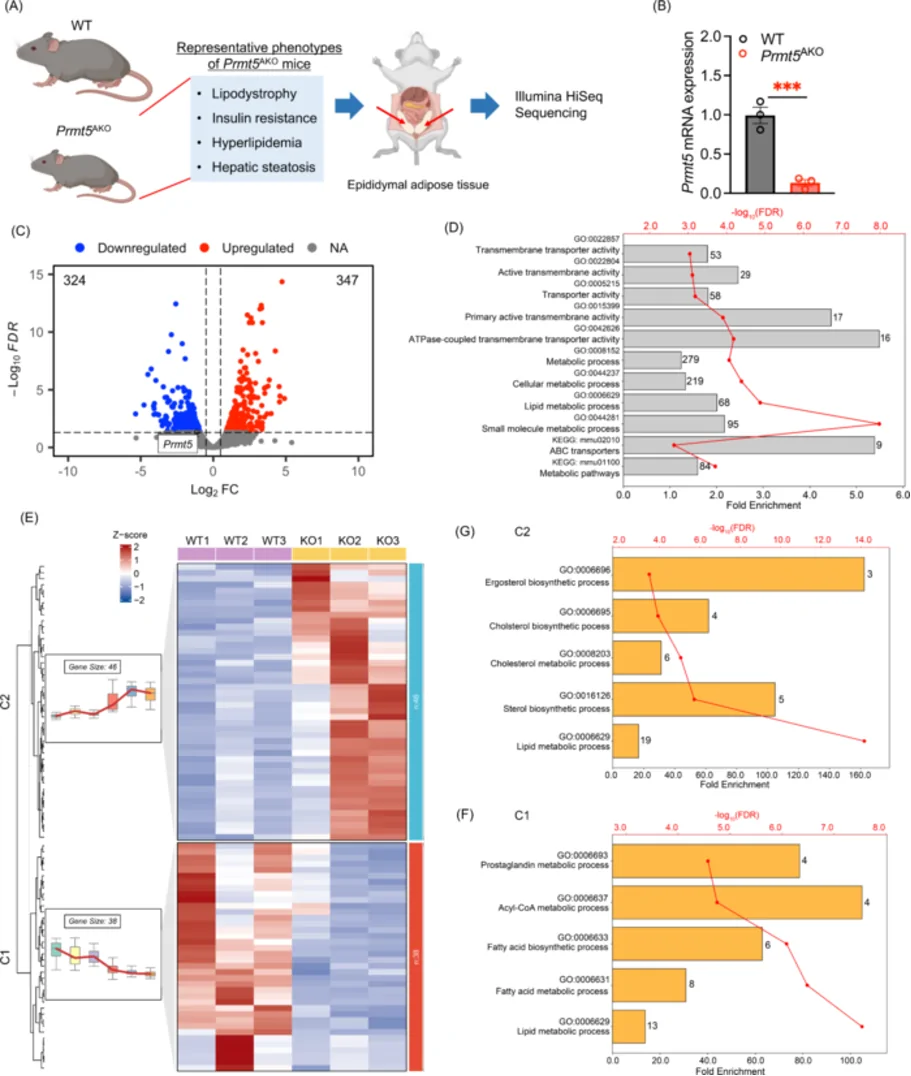
Summary of the major phenotypes of the *Prmt5* ^*AKO*^ mice and transcriptional profiling of white adipose tissue (WAT). (A) Phenotype of *Prmt5* ^*AKO*^ mice and experimental design. Epididymal WAT (eWAT) from WT and *Prmt5* ^*AKO*^ mice were used for RNA extraction and Illumina HiSeq sequencing. (B) Relative *Prmt5* mRNA levels in eWAT from WT and *Prmt5* ^*AKO*^ mice. ***p < 0.001. (C) Volcano plot of differentially expressed genes (DEGs) in eWAT from WT and *Prmt5* ^*AKO*^ mice. Log_2_ fold change (FC) in gene expression level in *Prmt5* ^*AKO*^ versus WT mice and the corresponding significance values displayed as −log_10_ false discovery rate (*FDR)*. The dashed line indicates the cutoff value for differential expression (*FDR* < 0.05 and |Log_2_FC| > 0.5). In total, 324 genes were downregulated (blue) and 347 genes were upregulated (red) by *Prmt5* ^AKO^. (D) Selected pathways significantly enriched by DEGs between *Prmt5* ^*AKO*^ and WT mice, based on annotation of Kyoto Encyclopedia of Genes and Genomes (KEGG) database and Gene Ontology (GO) term. Numbers labeled next to each bar represent the number of genes enriched in each term. Red lines represent the scaled −Log_10_ *FDR* value. (E) Heatmap of selected DEGs enriched in metabolic pathway, with specific metabolic pathway clustered into two groups (Cluster 1, C1, Cluster 2, C2). (F‐G) Top 5 GO terms enriched by each cluster from (E).

To investigate the role of PRMT5 in mature AT, we generated an adipocyte‐specific *Prmt5* knockout (*Prmt5* ^*AKO*^) mouse model. As summarized in Figure 1A, *Prmt5* ^*AKO*^ leads to progressive lipodystrophy, reduced energy expenditure, hyperlipidemia, hepatic steatosis, glucose intolerance, and insulin resistance [23]. Although both male and female *Prmt5* ^*AKO*^ mice showed reduced fat mass, the phenotype was more prominent in females, with a specific and pronounced reduction in epididymal WAT, a depot known to be more abundant in females [24]. Based on this stronger phenotype, we selected female mice for further studies.

To understand how *Prmt5* KO affects transcriptional pathways related to lipid metabolism, we performed RNA‐sequencing (RNA‐seq) using eWAT from 6‐month‐old WT and *Prmt5* ^*AKO*^ females via Illumina high‐throughput sequencing platform (Figure 1A). We confirmed the specific reduction of *Prmt5 mRNA* in the samples before library construction (Figure 1B).

In total, the RNA‐seq generated 27,081,232, 33,997,614, 34,890,502, 24,238,385, 30,244,956, 29,445,634 raw reads from the six (3 WT and 3 *Prmt5* ^*AKO*^) samples, which yielded 24,889,626, 30,159,890, 30,906,453, 21,983,924, 27,406,843, 26,615,776 high‐quality reads, respectively, with mapping rates ranging from 85.40% to 90.04% (Table S1). FastQC analysis demonstrated that over 20,000,000 raw reads from each sample had a mean sequence quality of 35 (Figure S1A), indicating good overall sequencing quality. We performed batch correction analysis to eliminate any artificial effects during the sample processing and sequencing. We applied false discovery rate (*FDR*) < 0.05 and |Log_2_ (fold change)| > 0.5 as cutoffs to identify differentially expressed genes (DEGs), resulting in a total of 671 DEGs (Table S2). These DEGs consisted of 347 upregulated genes and 324 downregulated genes, including *Prmt5*, in *Prmt5* ^*AKO*^ eWAT (Figure 1C). Genomic visualization also clearly showed the lack of target exon 7 in *Prmt5* ^*AKO*^ eWAT (Figure S1B).

### Loss of *Prmt5* alters the expression of genes related to metabolic and membrane transport pathways

To investigate the transcriptional changes in eWAT due to the deletion of *Prmt5*, we conducted functional enrichment analysis on DEGs and found that metabolic pathways and transport‐related pathways were significantly enriched in both Gene Ontology (GO) terms and Kyoto Encyclopedia of Genes and Genomes (KEGG) database (Figure 1D, Figure S1C,D, Tables S3–4). To gain more detailed insights into the dynamic changes, we performed the clustering trend analysis based on 84 DEGs enriched in KEGG metabolic pathway, and found these genes were clearly grouped into two clusters with varied response patterns towards *Prmt5* ^*AKO*^ (Figure 1E, Table S5). Cluster 1 contained 38 genes whose expression was decreased in the *Prmt5* ^*AKO*^ group, functionally enriched in FA and acyl‐CoA metabolic processes (Figure 1F, Table S5). Cluster 2 with 46 genes exhibited increased expression in the *Prmt5* ^*AKO*^ group, with functional enrichment of cholesterol and sterol metabolic process (Figure 1G, Table S5). Consistently, GO enrichment analysis demonstrated that DEGs were significantly enriched in several metabolic process pathways (Table S4), including FA metabolism (*Abcd2, Akr1cl, Acaa2, Acsm3, Akr1c18*), organic acid metabolism (*Errfl1*, *Abcd2*, *Acaa2*, *Acsm3*, *Gldc*), small molecule biosynthetic process (*Prps1, Errfl1, Akr1CL, Mvk, Acsm3*), and cholesterol homeostasis (*Errfl1, Dgat2, Abca5, Apoa2, Apoc3*).

**Figure 2:**
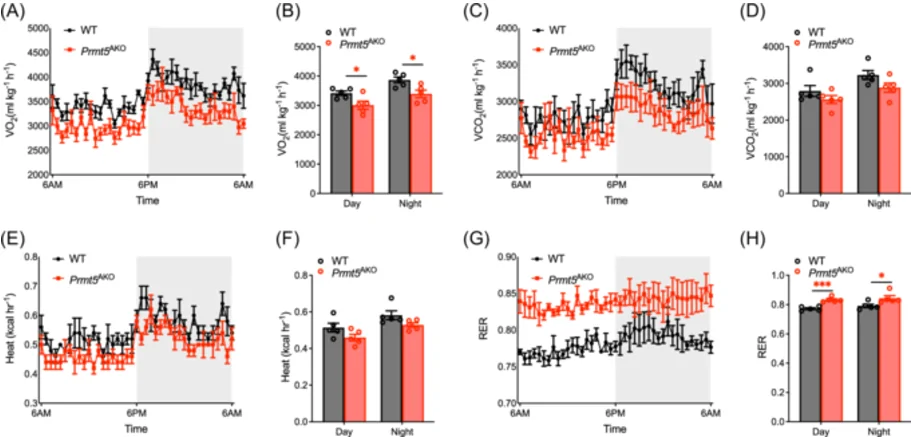
*Prmt5* ^*AKO*^ predominantly alters gene programs involved in metabolic pathways. (A) O_2_ consumption measured by indirect calorimetry and normalized to lean mass during a 48‐h cycle of 6‐month‐old WT and *Prmt5* ^*AKO*^ mice. (B) Average day and night O_2_ consumption of 6‐month‐old WT and *Prmt5* ^*AKO*^ mice, *n* = 5. *p < 0.05. (C) CO_2_ production measured by indirect calorimetry and normalized to lean mass during a 48‐h cycle of 6‐month‐old WT and *Prmt5* ^*AKO*^ mice. (D) Average day and night CO_2_ production of 6‐month‐old WT and *Prmt5* ^*AKO*^ mice, *n* = 5. (E) Heat production measured by indirect calorimetry and normalized to lean mass during a 48‐h cycle of 6‐month‐old WT and *Prmt5* ^*AKO*^ mice. (F) Average heat production of 6‐month‐old WT and *Prmt5* ^*AKO*^ mice, *n* = 5. (G) RER, a positive indicator of glucose utilization, measured by indirect calorimetry during a 48‐h cycle of 6‐month‐old WT and *Prmt5* ^*AKO*^ mice. (H) Average day and night RER of 6‐month‐old WT and *Prmt5* ^*AKO*^ mice, *n* = 5. *p < 0.05, ***p < 0.001.

We next examined whether the observed gene expression changes led to metabolic functional alterations. Using indirect calorimetry, we measured rates of oxygen (O_2_) consumption rate (OCR), carbon dioxide production (CO_2_), and heat production of 6‐month‐old WT and *Prmt5* ^*AKO*^ females. *Prmt5* ^*AKO*^ mice exhibited a lower OCR than WT mice during both day and night (Figure 2A,B). However, the two groups showed no significant difference in CO_2_ production (Figure 2C,D) and energy expenditure (Figure 2E,F).

We then calculated the respiratory exchange ratio (RER), which is defined as the ratio of CO_2_ produced to O_2_ consumed. RER level correlates with the predominant fuel sources used during metabolism, as an RER close to 0.7 suggests lipid oxidation, whereas an RER near 1.0 indicates carbohydrate oxidation. *Prmt5* ^*AKO*^ mice showed a significantly elevated RER compared to WT mice (Figure 2G,H), suggesting a metabolic shift from lipid oxidation to carbohydrate utilization.

### *Prmt5* deletion alters the expression profiles of membrane transporter genes

Among the significantly enriched pathways by DEGs, we found several of them were related to transport functions (Figure 1D). According to the GO enrichment, the top biological process (BP) enriched by DEGs were transport‐related pathways, including ATPase‐coupled transmembrane transporter activity, primary active transmembrane transporter activity, transporter activity (Figure S1E, Table S4). These results suggest a previously unrecognized role of PRMT5 in regulating membrane transport processes, which may have implications for AT metabolism and systemic metabolic homeostasis.

**Figure 3:**
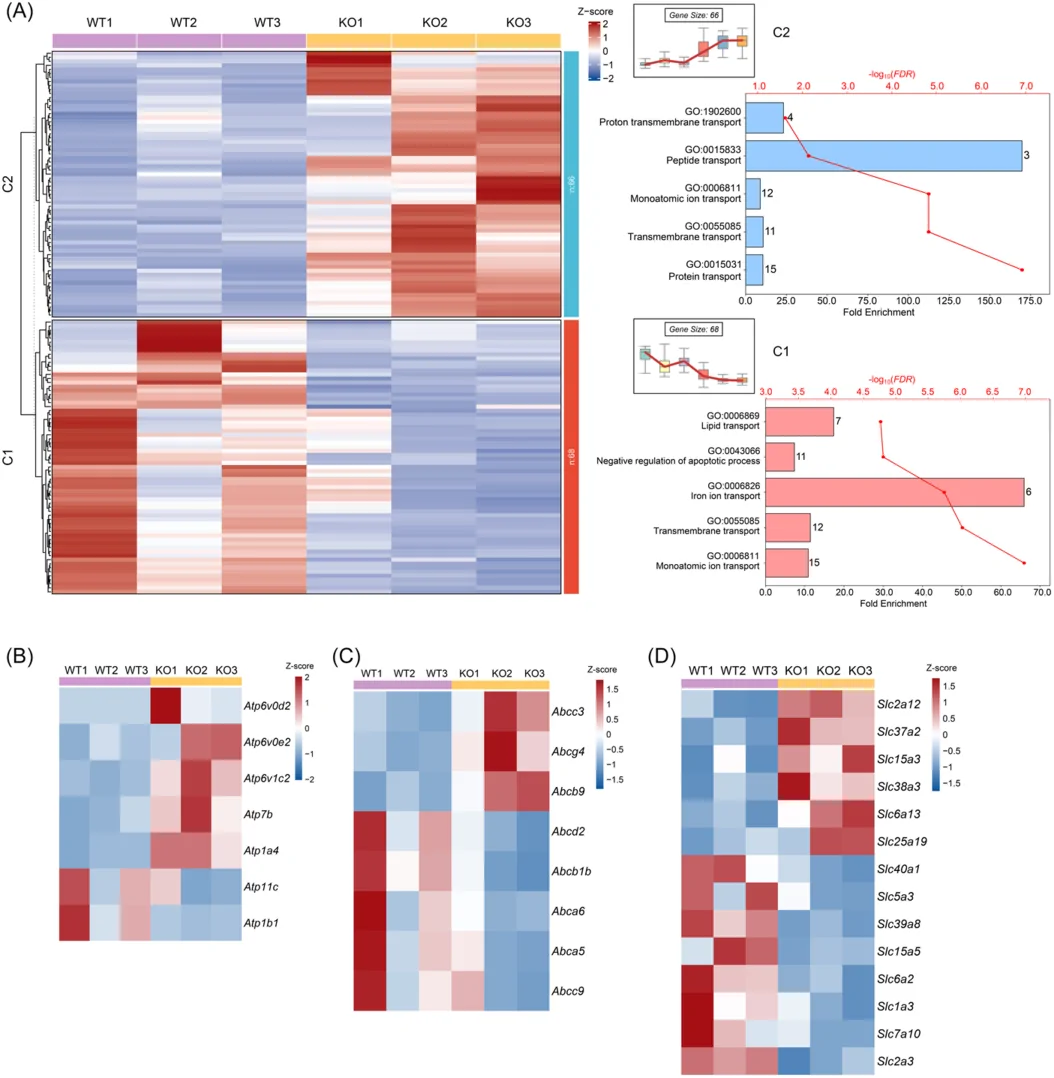
*Prmt5* ^*AKO*^ affects gene programs related to membrane transporters. (A) Heatmap of selected DEGs enriched in transport pathway, with specific metabolic pathway clustered into two groups (Cluster 1, C1, Cluster 2, C2). (B). Heatmap of DEGs involved in the ATPase family of transporters. (C) Heatmap of DEGs involved in the ABC family of transporters. (D) Heatmap of DEGs involved in the solute carrier family (SLC) family of transporters.

**Figure 4:**
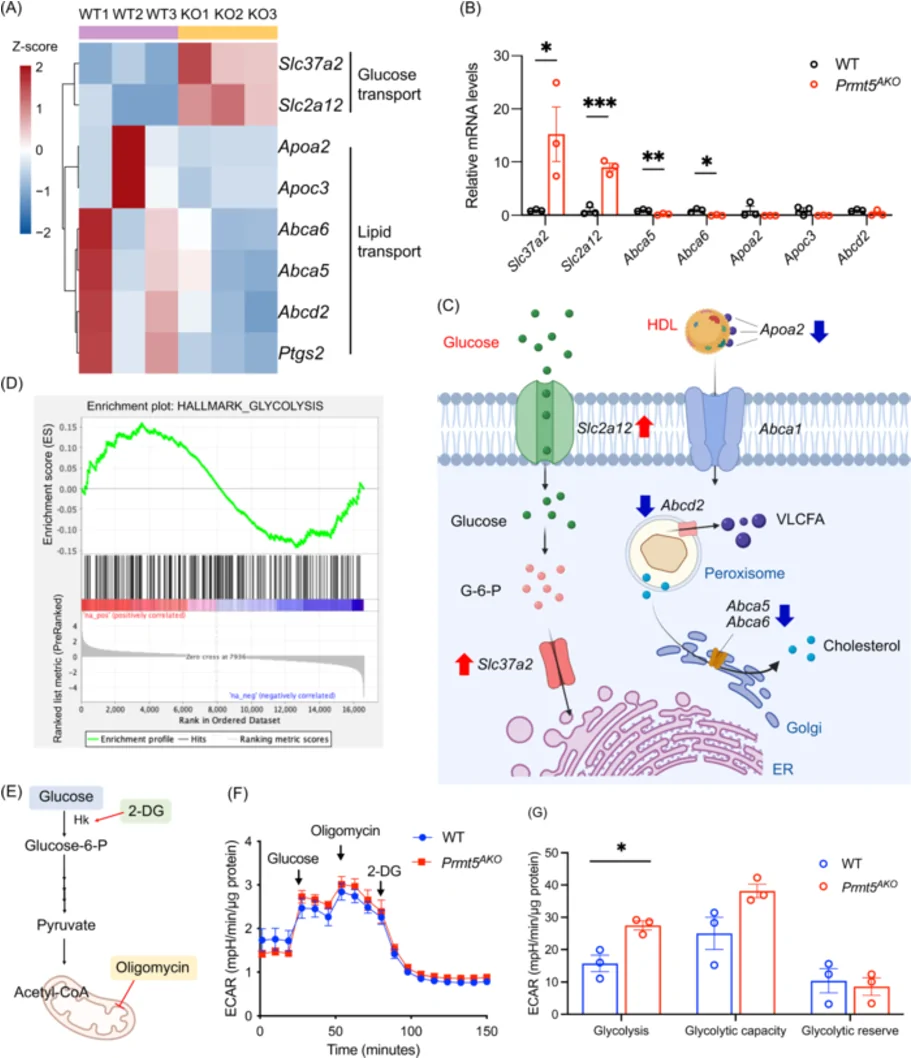
*Prmt5* ^*AKO*^ suppresses fatty acid transport, while promoting glucose transport and glycolysis. (A) Heatmap of selected DEGs involved in glucose and lipid transport. (B) Relative mRNA levels of selected genes involved in glucose and lipid transport in eWAT of WT and *Prmt5* ^*AKO*^ mice. *p < 0.05, **p < 0.01, ***p < 0.001. (C) Diagram showing the altered expression of genes involved in glucose and lipid transport in *Prmt5* ^*AKO*^ mice compared to WT mice. (D) Gene Set Enrichment Assay (GSEA) showing enrichment of genes related to “glycolysis” in the *Prmt5* ^*AKO*^ and WT mice. A positive value indicates correlation with *Prmt5* ^*AKO*^ phenotype, and negative value indicates correlation with WT phenotype. (E) Diagram showing the simplified glycolysis and the sites of action of the selected components. 2‐DG competes with glucose and inhibits glycolysis. Oligomycin inhibits ATP synthase in the mitochondria, resulting in increased glycolysis. (F) Seahorse measurement of extracellular acidification rate (ECAR) of adipocytes differentiated from *Prmt5* ^*AKO*^ and WT preadipocytes. (G) Quantification of glycolysis and glycolytic capacity based on ECAR, *n* = 3. *p < 0.05.

To gain further insight into how PRMT5 influences membrane transport pathways, we examined the gene profiles of 134 DEGs enriched in GO transport pathways and grouped them into two clusters (Figure 3A, Table S6). Cluster 1 with 68 genes showed reduced expression in *Prmt5* ^*AKO*^ group, with functional enrichment of monoatomic ion, iron ion, lipid, and sodium ion transport pathways. Cluster 2 with 66 genes exhibited increased expression in *Prmt5* ^*AKO*^ group, with functional enrichment of protein, monoatomic ion, peptide, proton, and carbohydrate transport pathways. These genes can be further classified based on their substrate specificity and mode of diffusion, including ATPase transporters, ABC transporters, and solute carrier family (SLC) transporters [25]. ATPase transporters, which use ATP hydrolysis to actively transport specific molecules, consist of four subtypes. Our analysis revealed that the expression levels of certain V‐type ATPases (encoded by *Atp6v0d2*, *Atp6v0e2*, *Atp6v1c2*), which are located in vacuoles such as acidic vesicles and lysosomes [26], were increased in *Prmt5* ^*AKO*^ eWAT (Figure 3B), consistent with our previous finding of the elevated autophagic flux in *Prmt5* ^*AKO*^ muscles [27]. In contrast, the expression of P‐type ATPases, which are localized in the plasma membrane, was either upregulated (*Atp1a4, Atp7b*) or downregulated (*Atp11c* and *Atp1b1*) (Figure 3B). ABC transporters contain an ATP‐binding domain and use ATP hydrolysis to transport various molecules across cell membranes [25]. We found the expression levels of *Abcg4*, *Abcb9*, and *Abcc3*, which function as cholesterol transporter, amino acid transporter, and multidrug transporter, respectively, were increased with *Prmt5* ^*AKO*^, while levels of *Abcd2*, *Abcb1b, Abca5*, and *Abca6*, involved in FA transport, were decreased (Figure 3C). Expression of genes from the SLC family exhibited a chaotic, random pattern of changes (Figure 3D). Specifically, upregulated genes were involved in glucose transport (*Slc2a12*, *Slc37a2*), membrane amino acid transport (*Slc15a3*, *Slc38a3*, *Slc6a13*) and mitochondrial substrate transport (*Slc25a19*), while downregulated genes were related to ion transport (*Slc40a1*, *Slc5a3*, *Slc39a8*), small peptide transport (*Slc15a5, Slc6a2*) and amino acid transport (*Slc1a3*, *Slc7a10*) (Figure 4A). The increase in *Slc37a2*, *Slc2a12*, and the decrease in *Abca5*, *Abca6* were validated by qPCR (Figure 4B). The upregulation of glucose transporters is consistent with the earlier observation of the increased carbohydrate diet utilization (RER) in the *Prmt5* ^*AKO*^ mice (Figure 2H). In addition to the three categories, downregulated genes, including *Apoa2*, *Apoa3*, *Gulp1*, *Pla2g4a*, *Ptgs2*, and *Ptgds* were associated with lipid and FA transport, while upregulated genes, such as *Mfsd12*, *Rtn2*, *Nup210*, *Timm13*, *Tap1*, *Ap3b2*, and *Arfgap1*, were related to protein, peptide, carbohydrate transport (Table S6). These results collectively suggest that loss of *Prmt5* leads to a general trend of decrease in lipid transport and increase in cholesterol and glucose transport (Figure 4C).

### *Prmt5* ^*AKO*^ causes fuel switch from lipid to glucose metabolism

We next analyzed how *Prmt5* ^*AKO*^ affects other pathways linked to glucose transport. Using Gene Set Enrichment Assay (GSEA) with Hallmark data set, we identified “glycolysis” in the upregulated pathways in *Prmt5* ^*AKO*^ WAs (Figure 4D, Table S7). We further investigated whether the upregulated glycolysis pathway genes lead to elevated anaerobic glycolysis in WAs. To this end, we used Seahorse bioanalyzer to measure the extracellular acidification rate (ECAR) of adipocytes differentiated from *Prmt5* ^*AKO*^ and WT preadipocytes isolated from eWATs (Figure 4E). Our results indicate that the ECAR associated with glycolysis after glucose challenge was significantly higher in *Prmt5* ^*AKO*^ than in WT adipocytes (Figure 4F,G). Our findings suggest that the improved glycolysis of *Prmt5*‐null WAs may have contributed to systemic diet switch towards glucose utilization in *Prmt5* ^*AKO*^ mice.

**Figure 5:**
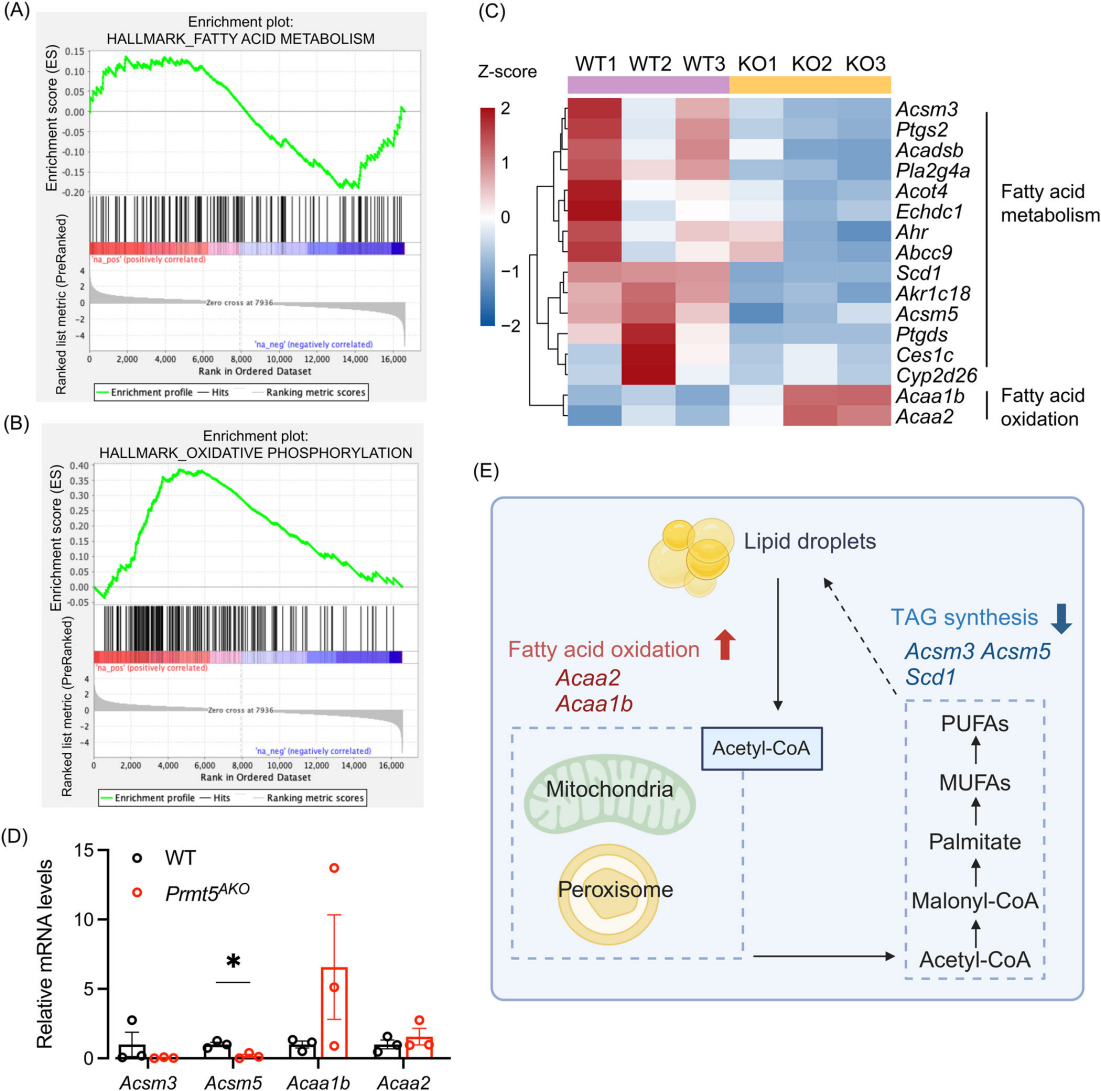
*Prmt5* ^*AKO*^ reduces fatty acid metabolism in WAT. (A) GSEA showing enrichment of genes related to fatty acid metabolic process in the *Prmt5* ^*AKO*^ and WT mice. A positive value indicates correlation with *Prmt5* ^*AKO*^ phenotype, and negative value indicates correlation with WT phenotype. (B) GSEA showing relative enrichment of oxidative phosphorylation in the *Prmt5* ^*AKO*^ and WT mice. A positive value indicates correlation with *Prmt5* ^*AKO*^ phenotype, and negative value indicates correlation with WT phenotype. (C) Heatmap of selected DEGs involved in FA metabolic process. (D) Relative mRNA levels of selected genes involved in FA metabolic process in eWAT of WT and *Prmt5* ^*AKO*^ mice, *n* = 3. *p < 0.05. (E) Diagram showing the FA metabolic process with genes significantly upregulated or downregulated in *Prmt5* ^*AKO*^ mice marked in red and blue, respectively.

We also analyzed downstream pathways of the lipid transport pathway downregulated in the *Prmt5* ^*AKO*^ WAs. The GSEA analysis showed that the “fatty acid metabolism” pathway was significantly downregulated in *Prmt5* ^*AKO*^ WAs (Figure 5A, Table S7). In contrast, DEGs enriched in the "oxidative phosphorylation"321 pathway showed a trend of being mostly upregulated in *Prmt5* ^*AKO*^, including *Cox8b* and *Cox7a1* (Figure 5B, Table S7). Specifically, *Prmt5* deletion significantly reduced the expression of TAG synthesis‐related genes, including *Acsm3*, *Acsm5*, and *Scd1* (Figure 5C,D). However, *Prmt5* deletion promoted the expression of *Acaa2* and *Acaa1b* (Figure 5C), which encoded proteins that catalyze mitochondrial and peroxisomal FA beta‐oxidation, respectively. Therefore, our findings suggest that the loss of *Prmt5* inhibits lipid transport and biosynthesis while promoting lipid oxidation (Figure 5E).

### Knockout of *Prmt5* leads to glycerophospholipid remodeling in WAs

**Figure 6:**
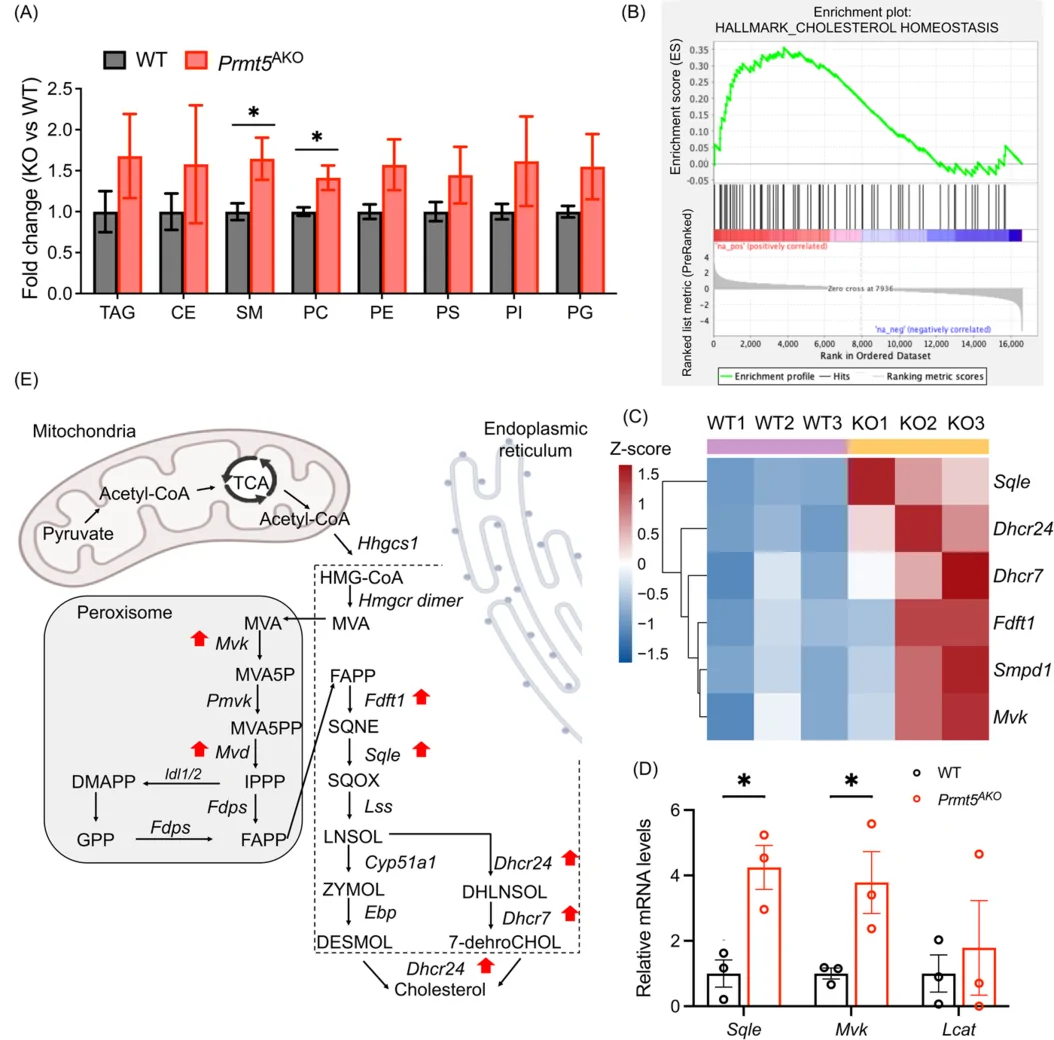
*Prmt5* ^*AKO*^ induces glycerophospholipid remodeling in WAT. (A) Fold change of the lipid species in eWAT from *Prmt5* ^*AKO*^ and WT mice. Lipid species include triacylglycerol (TAG), cholesteryl ester (CE), phosphatidylcholine (PC), phosphatidylethanolamine (PE), phosphatidylserine (PS), phosphatidylinositol (PI), phosphatidylglycerol (PG), and sphingomyelin (SM). *p < 0.05. (B) GSEA showing significant enrichment of genes involved in the “cholesterol homeostasis” pathway in the *Prmt5* ^*AKO*^ mice compared to WT mice. A positive value indicates correlation with *Prmt5* ^*AKO*^ phenotype, and negative value indicates correlation with WT phenotype. (C) Heatmap of DEGs involved in cholesterol metabolism. (D) Relative mRNA levels of selected genes involved in cholesterol metabolism in eWAT of WT and *Prmt5* ^*AKO*^ mice. *p < 0.05. (E) Peroxisome and cholesterol biogenesis processes with genes significantly upregulated in *Prmt5* ^*AKO*^ mice are marked by red arrows.

To understand how alterations in lipid metabolic gene expression affect lipid composition, we conducted a MS‐based lipidomics analysis on eWAT from 6‐month‐old *Prmt5* ^*AKO*^ and WT females. We identified more than 1084 differentially expressed lipid species belonging to 10 classes, including 190 TAGs, 56 CEs, 21 FFAs, 710 glycerophospholipids (PC, PE, PS, PI, phosphatidylglycerol (PG)), and 107 sphingolipids (Ceramide (Cer) and sphingomyelin (SM)) (Table S8). The principal component analysis (PCA) plot indicated fundamental differences between WT and *Prmt5* ^*AKO*^ groups manifested by two distinct clusters (Figure S2A). Further analysis revealed significant increases in the contents of PC and SM in the *Prmt5* ^*AKO*^ WAs (Figure 6A), suggestive of increased membrane curvature due to reduced size of *Prmt5*‐null WAs. However, there were no significant differences in other lipid classes. To further investigate the specific lipid species regulated by *Prmt5* ^*AKO*^, we visualized all significant changes in individual lipid species from all lipid classes using a bubble map (Figure S2B). With a cutoff of *p* < 0.05, we identified 106 significantly changed lipid species in eWAT from *Prmt5* ^*AKO*^ groups. Among these, several TAGs were found to be substantially reduced (Figure S2B). Interestingly, the most significantly increased lipid species mainly comprised of glycerophospholipids (PC, PE, and PI) and SM (Figure S2B). These findings suggest that *Prmt5* deletion induces considerable alternation in the composition and content of lipid species in eWAT, resulting in decreased amounts of TAGs and increased glycerophospholipids and SM.

We further analyzed the impact of *Prmt5* deletion on glycerophospholipids. We observed a significant increase in total PC levels in *Prmt5* ^*AKO*^ eWAT, with PCs containing C34:2 and C36:2 being the most upregulated (Figure S2C). Notably, most of the significantly upregulated glycerophospholipids, including PC, PE, and PI, contained at least one unsaturated carbon chain. Consistently, upregulated DEGs in *Prmt5* ^*AKO*^ eWAT were involved in glycerophospholipids biosynthesis (Table S4). Similarly, *Pla2g2e*, which regulates the internal conversion among different species of PL, was among the upregulated DEGs. Interestingly, DEGs involved in ether lipid metabolism, such as *Pla2g4e*, were significantly decreased in *Prmt5* ^*AKO*^ eWAT. These results suggest that PRMT5 is involved in the ether lipid metabolism pathways, and its deletion leads to glycerophospholipid remodeling, particularly an increase in PC levels.

Since glycerophospholipids are key components of membrane structure, we performed transmission electron microscopy (TEM) on eWAT from 6‐month‐old *Prmt5* ^*AKO*^ and WT females (Figure S3A). TEM analysis revealed ruffling of LD membrane and the presence of small vesicles in *Prmt5* ^*AKO*^ eWAT, which correlate with the observed PC increase. To further investigate whether elevated PC levels affect membrane structure, we performed NR12S staining to assess membrane fluidity in adipocytes. This dye can reflect the status of lipid packing via general polarization (GP) values, with higher GP values indicating more rigid membranes. Our results revealed that *Prmt5*‐deficient adipocytes exhibited significantly reduced membrane fluidity compared to WT adipocytes, as indicated by a higher GP value (Figure S3B). These results indicate *Prmt5* deletion leads to glycerophospholipid dynamics and membrane rigidification.

### Loss of *Prmt5* promotes cholesterol biosynthesis

Given the upregulation of cholesterol transport pathway in the *Prmt5* ^*AKO*^ WAs, we sought to investigate the impact of *Prmt5* loss on cholesterol biosynthesis pathways. GSEA using the Hallmark database indicated a significant upregulation of genes enriched in cholesterol homeostasis pathways such as *Sqle*, *Dhcr7*, and *Mvd* (Figure 6B, Table S7). Functional enrichment of cholesterol metabolism pathways also revealed the expression levels of *Dhcr24*, *Dhcr7*, *Fdft1*, and *Sqle*, which encode key proteins involved in the cholesterol biosynthesis pathways in ER, were upregulated after *Prmt5* knockout (Figure 6C,D). Moreover, the expression level of *Mvk* and *Smpd1*, which play critical roles in peroxisomal cholesterol biosynthesis pathways, was upregulated after *Prmt5* deletion (Figure 6C,D). Additionally, GSEA using the Hallmark database demonstrated that upregulated genes in *Prmt5* ^*AKO*^ were specifically enriched in peroxisome pathways (Table S7). However, *Lcat* gene, responsible for the formation of CE from free cholesterol, shows no difference from RNA‐seq data (Figure 6D, Figure S2D). Consistently, the total concentrations of CE were similar in WT and *Prmt5* ^*AKO*^ WAs (Figure 6A). Together, these results indicate that *Prmt5* ^*AKO*^ mice promote cholesterol biosynthesis in WAs and free cholesterol accumulation in the circulation (Figure 6E).

## 3 DISCUSSION

We have previously reported that the absence of *Prmt5* in adipocytes leads to age‐ and sex‐dependent lipodystrophy, accompanied by insulin resistance, hepatic steatosis, and systemic metabolic dysfunction [23]. These findings establish PRMT5 as a critical regulator of adipocyte biology. Nevertheless, the precise mechanism of PRMT5 in governing lipid dynamics remains unclear. In this study, we utilized unbiased RNA‐seq and MS‐based lipidomics to investigate the transcriptomic and lipidomic changes associated with the loss of *Prmt5* in WAs. Our aim was to provide an in‐depth molecular signature underlying PRMT5 function in WAs. This information may have potential implications for developing therapeutic interventions for metabolic disorders.

Our transcriptional analysis revealed significant gene reprogramming in *Prmt5* knockout mice, resulting in profound metabolic alterations and a systemic energy shift, along with the lipodystrophy and insulin‐resistant phenotype of *Prmt5* ^*AKO*^ mice. One of our key findings was a metabolic shift in substrate utilization from FAs to glucose in *Prmt5* ^*AKO*^ mice, as demonstrated by both transcriptional profiling and indirect calorimetry. The RER reflects the primary fuel source for metabolism, with carbohydrate oxidation (RER = 1) yielding more CO_2_ per O_2_ consumed than fat oxidation (RER = 0.7) [28]. Our results indicated that the average RER of *Prmt5* ^*AKO*^ mice was 0.82, whereas that of WT mice was 0.76. The higher RER level in *Prmt5* ^*AKO*^ mice suggests a shift towards more carbohydrate utilization. This was further supported by upregulation of glucose transport genes, *Slc2a12*, and *Slc37a2*, in AT of *Prmt5* ^*AKO*^ mice. Notably, while *Slc2a4* (GLUT4) did not show significant changes at mRNA levels, likely due to regulation through subcellular localization, as GLUT4 translocation from cytosolic vesicles to the cell membrane leads to elevated glucose uptake [29]. Conversely, the glucose transporter *Slc2a3* showed reduced expression due to its high glucose affinity. It has been reported that glucose transferred by GLUTs with lower glucose affinity would be more dependent on the circulating glucose concentration [30]. Since GLUT12 coded by *Slc2a12* has lower glucose affinity than GLUT3 coded by *Slc2a3*, GLUT12 might respond to the high blood glucose level more efficiently than GLUT3 in *Prmt5* ^*AKO*^ mice at 6 months old, suggesting it might be responsible for the increased glucose transport. Thus, changes in the genetic variation of transport pathway reinforced a possible switch from FA to glucose transport, which subsequently caused the fuel utilization change in *Prmt5* ^*AKO*^ eWAT. With higher level of *Slc2a12*, more glucose would enter the cytosol, while increased *Slc37a2*, known as glucose‐6‐phosphate transporter [31], led to the translocation of glucose into the lumen of the ER for hydrolysis. Importantly, these metabolic characteristics are commonly observed in human metabolic diseases such as T2D and obesity. Clinical studies have demonstrated that individuals with insulin resistance often exhibit impaired lipid oxidation and a compensatory reliance on glucose metabolism, with higher RER levels and impaired GLUTs [32, 33, 34]. Our study may therefore provide insights into how epigenetic regulator like PRMT5 contribute to metabolic diseases.

In parallel, we observed a downregulation of genes involved in FA metabolism and a corresponding reduction in TAGs and FFAs levels in *Prmt5* ^*AKO*^ eWAT. Specifically, *Acsm3* and *Acsm5* initiate cellular FA metabolism by converting FAs into their corresponding acyl‐CoA. Our findings are consistent with previous reports that PRMT5 methylated and stabilized SREBP1a, a key transcription factor in the regulation of lipid synthesis [23, 35]. Indeed, we observed a reduction in the expression levels of SREBP1 target genes, such as *Scd1*, in *Prmt5* ^*AKO*^ eWAT. Additionally, although PRMT5 has been reported to be essential for mitochondrial biogenesis and dynamics in hepatocytes and cancer cells [36, 37], the physiological role of PRMT5 in adipocyte mitochondria remains unknown. Interestingly, despite the increased expression of mitochondrial‐related genes, such as *Cox8b* and *Cox7a1*, and an elevated number of mitochondria observed by TEM in *Prmt5* ^*AKO*^ eWAT, these mice exhibited reduced energy expenditure. This seemingly paradoxical finding can be explained by impaired mitochondrial function. Our previous study demonstrated that WAs from *Prmt5* ^*AKO*^ mice exhibit significantly reduced OCR, as measured by Seahorse assay [23]. The upregulation of mitochondrial genes in the *Prmt5* ^AKO^ may be triggered by a feedback mechanism in response to reduced mitochondrial function. A similar phenotype has been reported in BSCL2‐deficient patients, where increased mitochondria with abnormal morphology coexist with impaired respiration [38]. In line with this, we previously showed that PRMT5 regulates *Bscl2* expression by methylating its transcription elongation factor SPT5 [23]. Therefore, loss of PRMT5 disrupts this regulation of *Bscl2*, potentially leading to mitochondrial abnormalities that similar with BSCL2‐deficient patients.

Serum analysis revealed that *Prmt5* knockout mice exhibited significantly higher levels of cholesterol, HDL, and LDL, which led to an overburdened liver developing into fatty liver [23]. In accordance, we observed increased expression of genes involved in cholesterol biogenesis in *Prmt5* ^*AKO*^ eWAT. Interestingly, lipidomics results showed no difference of CE content in eWAT. Consistent with this, transcriptome analysis showed no change in *Lcat* gene expression, which encodes the enzyme lecithin‐cholesterol acyltransferase, responsible for the formation of cholesteryl ester. These results suggest that *Prmt5* deletion promotes free cholesterol accumulation without affecting its esterification. Free cholesterol is essential for systemic metabolism, as it maintains cell membrane integrity and serves as a precursor for steroid hormones and vitamin D synthesis. Recent evidence suggests that AT participates in several metabolic activities, such as hormone secretion and cholesterol efflux, in addition to its energy storage function [39]. Therefore, it would be interesting to further investigate the proteomic analysis of the secretome and exosomes from *Prmt5* ^*AKO*^ eWAT to determine if free cholesterol or other potential adipokines and small molecules are involved in systemic regulation.

Lipidomics also revealed a highly dynamic remodeling process in glycerophospholipid species in *Prmt5* ^*AKO*^ eWAT. *Gpam*, which catalyzes an essential step in glycerophospholipid biosynthesis, was downregulated, and several genes involved in glycerophospholipids interconversion and turnover also showed differential expression. Although no uniform shift in all glycerophospholipid classes was observed, specific changes in individual subspecies suggested selective remodeling in response to *Prmt5* deficiency. Among these, PC, the predominant glycerophospholipid in mammalian cells and a key component of cellular and organelle membrane [40], was significantly increased, indicating remarkable organelle reprogramming. This is particularly relevant to defects in LD biogenesis as previously reported in *Prmt5* ^*AKO*^ adipocytes [23]. The functional consequences of increased PC levels revealed that *Prmt5*‐deficient adipocytes displayed reduced membrane fluidity. These findings suggest that increased PC accumulation is associated with membrane rigidification, which may impair key processes such as LD expansion, vesicle budding, and organelle communication. Notably, reduced membrane fluidity has been associated with metabolic disorders, like insulin resistance [41], which is consistent with the metabolic dysfunction observed in *Prmt5* ^*AKO*^ mice.

Glycerophospholipids are known to be transported through both vesicular and non‐vesicular trafficking pathways [42]. As ABC transporters are known to be involved [42], dynamic gene expression profiling of ABC‐related genes could be related to the inter‐organellar transport of glycerophospholipids. The interfacial behavior of oxidized glycerophospholipids differs depending on the unsaturated bond distribution [43]. As unsaturation levels increase in glycerophospholipids, oxidation causes the formation of smaller‐sized oxidation products cleaved from larger lipid molecules with truncated hydrocarbon residues, further facilitating vesicle formation by allowing for the necessary curvature and flexibility during the budding process [44]. Additionally, glycerophospholipid biosynthesis occurs primarily in the ER, where enzymes such as phosphatidylcholine synthase and phosphatidylethanolamine methyltransferase catalyze the stepwise assembly of phospholipid molecules from precursor molecules. The newly synthesized phospholipids are then transported to other organelles, including the Golgi apparatus, via vesicular trafficking pathways. Consistently, unsaturated glycerophospholipids are predominant among the increased glycerophospholipid species in *Prmt5* ^*AKO*^ eWAT, and TEM results showed a bundle of small vesicles in *Prmt5* ^*AKO*^ eWAT, suggesting active vesicular trafficking activities. However, to further study this phenomenon, experiments such as isolating different organelles for RNA and lipid profiling and Immunofluorescent staining of selected transporters need to be applied.

While our study provides an in‐depth analysis of transcriptomic and lipidomic changes in *Prmt5*‐deficient WAs, we realize that mRNA levels do not always correlate with protein abundance or activity. As proteins are the ultimate effectors of cellular function, incorporating proteomic analysis in the future would greatly enhance the understanding of PRMT5‐mediated regulation in adipocytes. Performing quantitative proteomics with a focus on isolated organelles, as well as on the extracellular vesicles and the secretome, is important to validate key modulators identified from our RNA‐seq results.

The mechanism by which PRMT5 regulates these genes remains to be fully elucidated. PRMT5 is distinct from well‐characterized transcriptional regulators of adipogenesis and lipid metabolism, such as PPARγ and SREBP1. While PPARγ and SREBP1 function as transcription factors that directly bind DNA to activate downstream gene expression, PRMT5 primarily exerts its effects through PTM. Specifically, PRMT5 methylates histone proteins like H4R3 and H3R2, and non‐histone protein SREBP1a to regulate lipogenesis [35, 45]. The dual function in both chromatin remodeling and protein stabilization underscores PRMT5's unique and multifaceted role in adipocyte metabolism. In the context of enhanced cholesterol biogenesis, we identified several binding peaks in the promoter regions of both *Dhcr7* and *Sqle*, by analyzing PRMT5 CHIP‐seq data from 3T3‐L1 adipocytes [46]. Thus, PRMT5 may directly regulate these loci, while future validation of the interaction is needed.

Another notable limitation of our study is that the analysis was conducted exclusively in females. The stronger phenotype in females allowed for enhanced sensitivity in detecting transcriptomic and lipidomic changes. However, given the known sex differences in adipose distribution, hormone signaling, and metabolic regulation, future studies are needed to assess whether the regulation of PRMT5 is conserved in male mice or exhibits sex‐specific differences.

Finally, our findings raise important considerations for the therapeutic use of PRMT5 inhibitors, which are currently in clinical trials for cancer. While PRMT5 inhibition is a promising therapy in oncology, our adipocyte‐specific knockout model revealed potential metabolic side effects, including elevated cholesterol biosynthesis, insulin resistance, and dyslipidemia. It is important to note that our results demonstrate the physiological consequences of PRMT5 deficiency at endogenous levels in adipocytes, which differ from the pathological overexpression often observed in cancer cells. Nevertheless, these findings highlight the need for caution in clinical application, as systemic PRMT5 inhibition may disrupt normal metabolic homeostasis. Therefore, therapeutic strategies must aim to target PRMT5 selectively in tumors but not completely abolish its expression in noncancerous tissues. To mitigate unintended metabolic effects, targeted delivery systems that restrict PRMT5 inhibition to tumor sites may be essential. Alternatively, co‐treatment approaches that preserve metabolic health could be explored.

## 4 CONCLUSION

Our study identifies PRMT5 as a key epigenetic regulator of adipocyte metabolism by modulating fuel preference and lipid homeostasis. Loss of PRMT5 disrupts multiple transcriptional processes, leading to systemic metabolic dysfunction, including insulin resistance and dyslipidemia. These findings broaden our understanding of adipose regulation and raise important considerations for the safe therapeutic targeting of PRMT5.

## 5 METHODS

### Animals

The Adipoq‐Cre (stock #010803) mice were obtained from Jackson Laboratory. The frozen sperms from mice harboring *Prmt5* ^*tm2c*^(EUCOMM)^wtsi^ were purchased from Wellcome Trust Sanger Institute. The in vitro fertilization, embryo development, and implantation in female C57BL/6 were performed in the Purdue University Transgenic and Genomic Editing Facility. PCR‐based genotyping was performed to screen for *Prmt5* ^*f/f*^ mice. The *Adipoq*‐Cre and *Prmt5* ^*flox/flox*^ mice were intercrossed for several generations to generate the *Prmt5* ^*AKO*^ mice as previously described [23]. *Prmt5* ^flox/flox^ mice were crossed with *Ucp1*‐Cre mice from Jackson Laboratory (stock#024670) to generate BA‐specific *Prmt5* KO mice (*Prmt5* ^UKO^ mice). The genotypes of experimental KO and associated control animals are as follows: *Prmt5* ^*AKO*^ (Adipoq‐Cre, *Prmt5* ^*flox/flox*^), *Prmt5* ^*UKO*^ (*Ucp1*‐Cre, *Prmt5* ^*flox/flox*^), and WT (*Prmt5* ^*flox/flox*^). Mice were housed in the animal facility with free access to water and standard rodent chow food (control diet) or HFD (TD.06414 Harlan). Mouse maintenance and experimental use were performed according to protocols approved by the Purdue Animal Care and Use Committee.

### Indirect calorimetry study

Oxygen consumption (VO_2_), carbon dioxide production (VCO_2_), RER, and heat production were measured by using an indirect calorimetry system (Oxymax, Columbus Instruments) installed under a constant environmental temperature (22°C) and a 12‐h light (06:00–18:00 h), 12‐h dark cycle (18:00–06:00 h). Mice in each chamber had free access to food (chow diet or HFD) and water. The raw data were normalized by body muscle mass, and the histograms of day (06:00–18:00 h) and night (18:00–06:00 h) values were the mean value of all points measured during the 12‐h period.

### Total RNA extraction and real‐time PCR

Total RNA was extracted from cells or tissues using Trizol Reagent according to the manufacturer's instructions. The purity and concentration of total RNA were measured by a spectrophotometer (Nanodrop 3000, Thermo Fisher) at 260 and 280 nm. All samples' absorption ratios (260/280 nm) were ~2.0. Then, 3 μg of total RNA were reversed transcribed using random primers and MMLV reverse transcriptase. Real‐time PCR was carried out with a Roche Lightcycler 480 PCR System using SYBR Green Master Mix and gene‐specific primers. Primer sequences were listed in Table S9. The 2^−ΔΔCT^ method was used to analyze the relative changes in gene expression normalized against mouse β‐Actin as an internal control.

### RNA‐seq analysis

Total RNA was extracted from eWAT of 6‐month‐old WT and *Prmt5* ^*AKO*^ female mice and subjected to RNA‐seq performed by Novo Bioinformatics Co., Ltd. Briefly, RNA quality analysis was checked by Agarose Gel Electrophoresis and Agilent 2100. A complementary DNA library was then constructed using mRNA enriched by anti‐polyA beads, and sequencing was performed according to the Illumina HiSeq standard protocol. Quality control for raw mRNA‐seq data was conducted using FastQC v0.11.9 [47]. Illumina adapter sequences and low‐quality bases were trimmed with Trim Galore v0.6.0 [48]. High‐quality paired‐end reads were then mapped to the mouse genome (GRCm38 vM25) using the STAR aligner v2.7.10a [49]. We utilized bam‐filter in ngsutilsj v0.5.9 to retain only properly and uniquely mapped paired reads (MAPQ ≥ 10) for downstream analysis [50]. Gene expression levels were summarized using FeatureCounts from the subread package v2.0.1 [51]. A differential expression analysis was performed using the EdgeR v4.0.16 [52]. The KEGG and GO pathway analyses were performed using the DAVID online software (https://davidbioinformatics.nih.gov/).

### Lipid extraction and targeted lipid profiling

Lipids were extracted from 4 to 6‐month‐old WT and *Prmt5* ^*AKO*^ mice using the Bligh & Dyer extraction method as described [53]. Briefly, 50 mg eWAT samples per mouse were transferred to a 2 mL vial with inert 1.4 mm ceramic (zirconium oxide) beads (Precellys CK 14, Bertin Corp, part # P000912‐LYSK0A), and 500 μL of ultrapure water was added to homogenize the sample using the Precellys tissue homogenizer (Bertin Corp) at three cycles of 6200 rpm for 20 s. Next, 200 μL of homogenized tissue was transferred to a new microtube and mixed with 250 μL chloroform and 450 μL of methanol. This solution was incubated at room temperature for 15 min. After that, 250 μL of chloroform and 250 μL of water were added, and the sample was centrifuged for 10 min at 16,000 × *g*, forming a 2‐phase solution where the bottom phase contained the lipids (organic phase). The organic phase was transferred to a new tube and dried using a speed vac centrifuge (Savant Speedvac, Thermo Scientific Inc.), and samples were stored at −80°C until mass spectrometry analysis.

Targeted lipid profiling was performed using discovery multiple reaction monitoring (MRM) profiling methods and instrumentation in the Metabolite Profiling Facility at Purdue University for MRM profiling as described [53, 54, 55]. Dried lipid extracts were diluted in 500 μL of methanol/chloroform 3:1 (v/v) (stock solution). The stock solution was further diluted 50× in injection solvent (acetonitrile/methanol/ammonium acetate 300 mM 3:6.65:0.35 (v/v)), and 8 μL of this solution was used for the profiling analysis of each lipid class using a micro‐autosampler (G1377A) to the ESI source of an Agilent 6410 triple quadrupole mass spectrometer (Agilent Technologies). A capillary pump was connected to the autosampler and operated at a flow rate of 7 μL/min and a pressure of 150 bar. The capillary voltage on the instrument was 3.5–5 kV, and the gas flow was 5.1 L/min at 300°C.

For the MS analysis, relative amounts of ion abundances were used for statistics. For the relative expression level, values of ion intensities for each MRM monitored were normalized by the total ion intensity of all MRMs in the method for a given sample. For absolute quantification, each injection was normalized to 16 μg of tissue used for the analysis based on the ion intensities of internal standards. Further statistical analysis was then performed using Metaboanalyst 4.0 software (https://www.metaboanalyst.ca). Uploaded data was auto‐scaled and analyzed with Student's *t*‐test with a two‐tailed distribution. Volcano plots (Fold change threshold 2, *p*‐value threshold 0.05), PCAs, and heatmaps were plotted. Fold change was calculated by dividing values of ion intensities for each of the MRMs measured in each sample by the ion intensity of the corresponding MRM in the blank.

### Seahorse XF cell glycolysis stress analysis

The glycolytic capacity was determined using the Agilent Seahorse XF Glycolysis Stress Test Kit (Agilent Technologies, 103020‐100). The cells were seeded in an XFe24 cell culture microplate at a density of 4 × 10^4^ cells/well. The Seahorse XFe24 Flux sensor cartridge was hydrated overnight in a utility plate filled with 800 μL of Seahorse Calibrant in a non‐CO_2_ incubator at 37°C. The next day, cells were exposed to Seahorse XF base medium supplemented with 1 mM l‐glutamine in a non‐CO_2_ incubator for 30 min before the assay. The ECAR was measured by the sequential injection of 100 μM glucose, 30 μM oligomycin, and 500 μM 2‐DG. After the experiment, cells were fixed in 20% (w/v) TCA overnight at 4°C. The fixed cells were washed four times with ddH_2_O and air‐dried at room temperature. Cells were stained with 0.04% sulforhodamine B (SRB) solution at room temperature for 1 h and quickly rinsed with 1% (v/v) acetic acid, followed by air drying. Cell lysates were resolved in 10 mM Tris‐base solution (pH 10.5), and the absorbance was measured at 510 nm to determine cell number. ECAR values were normalized to the number of cells in each well.

### Transmission electron microscopy

The eWAT from 6‐month‐old WT and *Prmt5* ^*AKO*^ female mice were cut into 1 × 2 mm blocks in a 6‐cm culture dish immediately after euthanizing the mice, and then fixed in 2.5% glutaraldehyde for 30 min. The blocks were continued to be fixed in 2.5% glutaraldehyde for 1 h, followed by fixation in 2% osmium tetroxide for 1 h. All the fixatives were made with 0.1 M cacodylate buffer. After washing, the blocks were dehydrated in a graded ethanol series and then embedded in Epon Generic Resin. The sections with a thickness of about 90 nm were prepared with uranyl acetate and lead citrate stain and examined with a transmission electron microscope (Gatan Digital Microscopy).

### Membrane fluidity measurement

Membrane fluidity was measured using the fluorescent lipids probe NR12S (Tocris, 7509). Mature adipocytes were isolated from adipose tissues and stained with 100 nM of NR12S for 30 min. After washing with PBS for three times, cells were analyzed using a spectral meter, with excitation wavelength at 554 nm and emission wavelengths set at 560 and 630 nm. Generalized polarization (GP) values were calculated using the formula: GP = (*I*_560_ − *I*_630_)/(*I*_560_ + *I*_630_).

### Statistical analysis

Trial experiments or experiments done previously were used to determine sample size with adequate statistical power. The researchers involved in the in vivo treatments were not completely blinded, but all insulin tolerance test and glucose tolerance test were conducted blindly. All images were randomly captured from the sample and analyzed in a blinded manner. No data were excluded from the following statistical analysis. All analyses were conducted with Student's *t*‐test with a two‐tailed distribution, and the graphs were made using GraphPad Prism software. All experimental data are represented as mean ± s.e.m (*n* ≥ 3). Comparisons with *p* values < 0.05 were considered statistically significant. Due to small sample size, normality could not be formally tested; data were assumed to be approximately normally distributed as commonly accepted in similar studies.

## AUTHOR CONTRIBUTIONS

**Shihuan Kuang** and **Zhihao Jia**: Conceptualization; methodology. **Xiyue Chen, Xiashiyao Zhang, Zhihao Jia**, and **Christina R. Ferreira**: Investigation. **Xiyue Chen, Xiashiyao Zhang, Zhihao Jia, Feng Yue, Jun Wan, James F. Markworth**, and **Christina R. Ferreira**: Data curation; formal analysis. **Xiyue Chen, Zhihao Jia, Xiashiyao Zhang, Jun Wan**, and **Shihuan Kuang**: Writing—original draft. **Shihuan Kuang, Zhihao Jia, Xiyue Chen, Xiashiyao Zhang, Christina R. Ferreira, Feng Yue, Jun Wan**, and **James F. Markworth**: Writing – review & editing; final manuscript approval. All authors have read the final manuscript and approved it for publication.

## CONFLICT OF INTEREST STATEMENT

The authors declare no conflicts of interest.

## ETHICS STATEMENT

Mouse maintenance and experimental use were performed according to protocols approved by the Purdue Animal Care and Use Committee (IPROTO1112000440).

## Supporting information


**Figure S1.** *Prmt5* ^*AKO*^ induces gene programs involved in metabolic and transport pathways.
**Figure S2.** *Prmt5* ^*AKO*^ induces changes in lipid dynamics.
**Figure S3.** *Prmt5* ^*AKO*^ alters membrane morphology in eWAT.

**Table S1.** Summary of RNA‐seq Reads and Mapping Statistics.
**Table S2.** Counts per million (CPM) values of all genes analyzed with edgeR.
**Table S3.** Pathway analysis of differentially expressed genes (DEGs) based on the KEGG database.
**Table S4.** Pathway analysis of differentially expressed genes (DEGs) based on the GO terms.
**Table S5.** Clustering and pathway analysis of metabolism‐related DEGs.
**Table S6.** Clustering and pathway analysis of transport‐related DEGs.
**Table S7.** Gene Set Enrichment Assay (GSEA) with Hallmark dataset.
**Table S8.** Lipidomics results.
**Table S9.** Primers for real‐time PCR.

## Data Availability

The data supporting this publication are available in the GSE290506 repository (https://www.ncbi.nlm.nih.gov/geo/query/acc.cgi?acc=GSE290506). The data and scripts used are saved in GitHub (https://github.com/xiyueccchen/IMO-2025.git). Supplementary materials (figures, tables, graphical abstract, slides, videos, Chinese translated version, and update materials) may be found in the online DOI or iMetaOmics http://www.imeta.science/imetaomics/.

## References

[1] Huh, Jin Young , Yoon Jeong Park , Mira Ham , and Jae Bum Kim . 2014. “Crosstalk Between Adipocytes and Immune Cells in Adipose Tissue Inflammation and Metabolic Dysregulation in Obesity.” Molecules and Cells 37: 365–371. 10.14348/molcells.2014.0074 PMC4044307 24781408

[2] Luo, Liping , and Meilian Liu . 2016. “Adipose Tissue in Control of Metabolism.” Journal of Endocrinology 231: R77–R99. 10.1530/JOE-16-0211 PMC7928204 27935822

[3] Birsoy, Kıvanç , William T. Festuccia , and Mathieu Laplante . 2013. “A Comparative Perspective on Lipid Storage in Animals.” Journal of Cell Science 126: 1541–1552. 10.1242/jcs.104992 23658371

[4] Townsend, Kristy L. , and Yu‐Hua Tseng . 2014. “Brown Fat Fuel Utilization and Thermogenesis.” Trends in Endocrinology & Metabolism 25: 168–177. 10.1016/j.tem.2013.12.004 PMC3972344 24389130

[5] Hausman, D. B. , M. DiGirolamo , T. J. Bartness , G. J. Hausman , and R. J. Martin . 2001. “The Biology of White Adipocyte Proliferation.” Obesity Reviews 2: 239–254. 10.1046/j.1467-789X.2001.00042.x 12119995

[6] Arisawa, K. , I. Ichi , Y. Yasukawa , Y. Sone , and Y. Fujiwara . 2013. “Changes in the Phospholipid Fatty Acid Composition of the Lipid Droplet during the Differentiation of 3T3‐L1 Adipocytes.” Journal of Biochemistry 154: 281–289. 10.1093/jb/mvt051 23760554

[7] Glatz, Jan F. C. , Joost J. F. P. Luiken , and Arend Bonen . 2010. “Membrane Fatty Acid Transporters as Regulators of Lipid Metabolism: Implications for Metabolic Disease.” Physiological Reviews 90: 367–417. 10.1152/physrev.00003.2009 20086080

[8] Epps, Caleb T. , Robin D. Clugston , Amit Saha , William S. Blaner , and Li‐Shin Huang . 2016. “Chapter 6 ‐ The Role of CD36 in the Pathogenesis of Alcohol‐Related Disease.” In Molecular Aspects of Alcohol and Nutrition (pp. 71–84). Academic Press. 10.1016/B978-0-12-800773-0.00006-9

[9] Kazantzis, Melissa , and Andreas Stahl . 2012. “Fatty Acid Transport Proteins, Implications in Physiology and Disease.” Biochimica et Biophysica Acta (BBA) ‐ Molecular and Cell Biology of Lipids 1821: 852–857. 10.1016/j.bbalip.2011.09.010 PMC3274620 21979150

[10] Bartz, René , Wen‐Hong Li , Barney Venables , John K. Zehmer , Mary R. Roth , Ruth Welti , Richard G. W. Anderson , Pingsheng Liu , and Kent D. Chapman . 2007. “Lipidomics Reveals That Adiposomes Store Ether Lipids and Mediate Phospholipid Traffic.” Journal of Lipid Research 48: 837–847. 10.1194/jlr.M600413-JLR200 17210984

[11] Chitraju, Chandramohan , Martin Trötzmüller , Jürgen Hartler , Heimo Wolinski , Gerhard G. Thallinger , Achim Lass , Rudolf Zechner , et al. 2012. “Lipidomic Analysis of Lipid Droplets From Murine Hepatocytes Reveals Distinct Signatures for Nutritional Stress.” Journal of Lipid Research 53: 2141–2152. 10.1194/jlr.M028902 PMC3435547 22872753

[12] Penno, Anke , Gregor Hackenbroich , and Christoph Thiele . 2013. “Phospholipids and Lipid Droplets.” Biochimica et Biophysica Acta (BBA) ‐ Molecular and Cell Biology of Lipids 1831: 589–594. 10.1016/j.bbalip.2012.12.001 23246574

[13] Calzada, Elizabeth , Ouma Onguka , and Steven M. Claypool . 2016. “Chapter Two ‐ Phosphatidylethanolamine Metabolism in Health and Disease.” International Review of Cell and Molecular Biology 321: 29–88. 10.1016/bs.ircmb.2015.10.001 PMC4778737 26811286

[14] Ridgway, N. D. , and D. E. Vance . 1987. “Purification of Phosphatidylethanolamine N‐Methyltransferase From Rat Liver.” Journal of Biological Chemistry 262: 17231–17239. 10.1016/S0021-9258(18)45514-7 3680298

[15] Bedford, Mark T. , and Steven G. Clarke . 2009. “Protein Arginine Methylation in Mammals: Who, What, and Why.” Molecular Cell 33: 1–13. 10.1016/j.molcel.2008.12.013 PMC3372459 19150423

[16] Yang, Yanzhong , and Mark T. Bedford . 2013. “Protein Arginine Methyltransferases and Cancer.” Nature Reviews Cancer 13: 37–50. 10.1038/nrc3409 23235912

[17] Karkhanis, Vrajesh , Yu‐Jie Hu , Robert A. Baiocchi , Anthony N. Imbalzano , and Saïd Sif . 2011. “Versatility of PRMT5‐Induced Methylation in Growth Control and Development.” Trends in Biochemical Sciences 36: 633–641. 10.1016/j.tibs.2011.09.001 PMC3225484 21975038

[18] Stopa, Nicole , Jocelyn E. Krebs , and David Shechter . 2015. “The PRMT5 Arginine Methyltransferase: Many Roles in Development, Cancer and Beyond.” Cellular and Molecular Life Sciences 72: 2041–2059. 10.1007/s00018-015-1847-9 PMC4430368 25662273

[19] Li, Zhenhua , Jingping Xu , Yao Song , Chong Xin , Lantao Liu , Ning Hou , Yan Teng , et al. 2021. “PRMT5 Prevents Dilated Cardiomyopathy via Suppression of Protein O‐GlcNAcylation.” Circulation Research 129: 857–871. 10.1161/CIRCRESAHA.121.319456 34503365

[20] Tsai, Wen‐Wei , Sherry Niessen , Naomi Goebel , John R. Yates , Ernesto Guccione , and Marc Montminy . 2013. “PRMT5 Modulates the Metabolic Response to Fasting Signals.” Proceedings of the National Academy of Sciences 110: 8870–8875. 10.1073/pnas.1304602110 PMC3670393 23671120

[21] Ma, Jian , Xin He , Yan Cao , Kienan O'Dwyer , Katherine M. Szigety , Yuan Wu , Buddha Gurung , et al. 2020. “Islet‐Specific Prmt5 Excision Leads to Reduced Insulin Expression and Glucose Intolerance in Mice.” Journal of Endocrinology 244: 41–52. 10.1530/JOE-19-0268 PMC6864278 31539871

[22] LeBlanc, Scott E. , Silvana Konda , Qiong Wu , Yu‐Jie Hu , Christine M. Oslowski , Saïd Sif , and Anthony N. Imbalzano . 2012. “Protein Arginine Methyltransferase 5 (Prmt5) Promotes Gene Expression of Peroxisome Proliferator‐Activated Receptor γ2 (PPARγ2) and Its Target Genes During Adipogenesis.” Molecular Endocrinology 26: 583–597. 10.1210/me.2011-1162 PMC3327358 22361822

[23] Jia, Zhihao , Feng Yue , Xiyue Chen , Naagarajan Narayanan , Jiamin Qiu , Sabriya A. Syed , Anthony N. Imbalzano , et al. 2020. “Protein Arginine Methyltransferase PRMT5 Regulates Fatty Acid Metabolism and Lipid Droplet Biogenesis in White Adipose Tissues.” Advanced Science 7: 2002602. 10.1002/advs.202002602 PMC7709973 33304767

[24] Palmisano, Brian T. , Lin Zhu , Robert H. Eckel , and John M. Stafford . 2018. “Sex Differences in Lipid and Lipoprotein Metabolism.” Molecular Metabolism 15: 45–55. 10.1016/j.molmet.2018.05.008 PMC6066747 29858147

[25] Hediger, Matthias A. , Benjamin, Clémençon , Robert E. Burrier , and Elspeth A. Bruford . 2013. “The ABCs of Membrane Transporters in Health and Disease (SLC Series): Introduction.” Molecular Aspects of Medicine 34: 95–107. 10.1016/j.mam.2012.12.009 PMC3853582 23506860

[26] Martina, Jose A. , and Rosa Puertollano . 2013. “RRAG GTPases Link Nutrient Availability to Gene Expression, Autophagy and Lysosomal Biogenesis.” Autophagy 9: 928–930. 10.4161/auto.24371 PMC3672304 23524842

[27] Kim, Kun Ho , Stephanie N. Oprescu , Madigan M. Snyder , Aran Kim , Zhihao Jia , Feng Yue , and Shihuan Kuang . 2023. “PRMT5 Mediates FoxO1 Methylation and Subcellular Localization to Regulate Lipophagy in Myogenic Progenitors.” Cell Reports 42: 113329. 10.1016/j.celrep.2023.113329 PMC10727913 37883229

[28] Farinatti, Paulo , Antonio G. Castinheiras Neto , and Paulo R. S. Amorim . 2016. “Oxygen Consumption and Substrate Utilization During and After Resistance Exercises Performed with Different Muscle Mass.” International Journal of Exercise Science 9: 77–88. 10.70252/AKBM3973 PMC4882463 27293507

[29] Olson, A. L. , and J. E. Pessin . 1996. “Structure, Function, and Regulation of the Mammalian Facilitative Glucose Transporter Gene Family.” Annual Review of Nutrition 16: 235–256. 10.1146/annurev.nu.16.070196.001315 8839927

[30] Day, P. E. , J. K. Cleal , E. M. Lofthouse , M. A. Hanson , and R. M. Lewis . 2013. “What Factors Determine Placental Glucose Transfer Kinetics?” Placenta 34: 953–958. 10.1016/j.placenta.2013.07.001 PMC3776928 23886770

[31] Wang, Y. , Y. Nakagawa , L. Liu , W. Wang , X. Ren , A. Anghel , K. Lutfy , T. C. Friedman , and Y. Liu . 2011. “Tissue‐Specific Dysregulation of Hexose‐6‐Phosphate Dehydrogenase and Glucose‐6‐Phosphate Transporter Production in db/db Mice as a Model of Type 2 Diabetes.” Diabetologia 54: 440–450. 10.1007/s00125-010-1956-9 PMC3795617 21052977

[32] Galgani, Jose E. , Cedric Moro , and Eric Ravussin . 2008. “Metabolic Flexibility and Insulin Resistance.” American Journal of Physiology‐Endocrinology and Metabolism 295: E1009–E1017. 10.1152/ajpendo.90558.2008 PMC2584808 18765680

[33] Matsuzaka, Takashi , and Hitoshi Shimano . 2012. “GLUT12: A Second Insulin‐Responsive Glucose Transporters as an Emerging Target for Type 2 Diabetes.” Journal of Diabetes Investigation 3: 130–131. 10.1111/j.2040-1124.2011.00177.x PMC4020729 24843555

[34] Abel, E. Dale , Odile Peroni , Jason K. Kim , Young‐Bum Kim , Olivier Boss , Ed Hadro , Timo Minnemann , Gerald I. Shulman , and Barbara B. Kahn . 2001. “Adipose‐Selective Targeting of the GLUT4 Gene Impairs Insulin Action in Muscle and Liver.” Nature 409: 729–733. 10.1038/35055575 11217863

[35] Liu, Liu , Xiaoping Zhao , Li Zhao , Jiajin Li , Hao Yang , Zongping Zhu , Jianjun Liu , and Gang Huang . 2016. “Arginine Methylation of SREBP1a via PRMT5 Promotes De Novo Lipogenesis and Tumor Growth.” Cancer Research 76: 1260–1272. 10.1158/0008-5472.CAN-15-1766 26759235

[36] Hinterschied, Claire , Fiona Brown , Janani Ravikrishnan , JoBeth Helmig‐Mason , Kaylee R. Harrison , Aneeq Yasin , Shelby Sloan , et al. 2022. “PRMT5 Inhibition Alters Mitochondrial Dynamics in Mantle Cell Lymphoma Creating Vulnerability to BH3 Mimetic Compounds.” Blood 140: 3118–3119. 10.1182/blood-2022-167712

[37] Huang, Lei , Jehnan Liu , Xiao‐Ou Zhang , Katelyn Sibley , Sonia M. Najjar , Mary M. Lee , and Qiong Wu . 2018. “Inhibition of Protein Arginine Methyltransferase 5 Enhances Hepatic Mitochondrial Biogenesis.” Journal of Biological Chemistry 293: 10884–10894. 10.1074/jbc.RA118.002377 PMC6052201 29773653

[38] Combot, Yoann , Veijo T. Salo , Gilliane Chadeuf , Maarit Hölttä , Katharina Ven , Ilari Pulli , Simon Ducheix , et al. 2022. “Seipin Localizes at Endoplasmic‐Reticulum‐Mitochondria Contact Sites to Control Mitochondrial Calcium Import and Metabolism in Adipocytes.” Cell Reports 38: 110213. 10.1016/j.celrep.2021.110213 35021082

[39] Zhang, Tianhua , Jin Chen , Xiaoyu Tang , Qin Luo , Danyan Xu , and Bilian Yu . 2019. “Interaction Between Adipocytes and High‐Density Lipoprotein: New Insights Into the Mechanism of Obesity‐Induced Dyslipidemia and Atherosclerosis.” Lipids in Health and Disease 18: 223. 10.1186/s12944-019-1170-9 PMC6913018 31842884

[40] Petkevicius, Kasparas , Sam Virtue , Guillaume Bidault , Benjamin Jenkins , Cankut Çubuk , Cecilia Morgantini , Myriam Aouadi , et al. 2019. “Accelerated Phosphatidylcholine Turnover in Macrophages Promotes Adipose Tissue Inflammation in Obesity.” eLife 8: e47990. 10.7554/eLife.47990 PMC6748830 31418690

[41] Desai, Aditya J. , and Laurence J. Miller . 2018. “Changes in the Plasma Membrane in Metabolic Disease: Impact of the Membrane Environment on G Protein‐Coupled Receptor Structure and Function.” British Journal of Pharmacology 175: 4009–4025. 10.1111/bph.13943 PMC6177615 28691227

[42] Yang, Yanbo , Minhyoung Lee , and Gregory D. Fairn . 2018. “Phospholipid Subcellular Localization and Dynamics.” Journal of Biological Chemistry 293: 6230–6240. 10.1074/jbc.R117.000582 PMC5925819 29588369

[43] Rudolphi‐Skórska, Elżbieta , Maria Filek , and Maria Zembala . 2017. “The Effects of the Structure and Composition of the Hydrophobic Parts of Phosphatidylcholine‐Containing Systems on Phosphatidylcholine Oxidation by Ozone.” The Journal of Membrane Biology 250: 493–505. 10.1007/s00232-017-9976-8 PMC5613038 28799139

[44] Campbell, Robert B. , Sathyamangalam V. Balasubramanian , and Robert M. Straubinger . 2001. “Phospholipid‐Cationic Lipid Interactions: Influences on Membrane and Vesicle Properties.” Biochimica et Biophysica Acta (BBA) ‐ Biomembranes 1512: 27–39. 10.1016/S0005-2736(01)00290-5 11334622

[45] Strahl, Brian D. , and C. David Allis . 2000. “The Language of Covalent Histone Modifications.” Nature 403: 41–45. 10.1038/47412 10638745

[46] Syed, Sabriya A. , Kristina Shqillo , Ankita Nand , Ye Zhan , Job Dekker , and Anthony N. Imbalzano . 2023. “Protein Arginine Methyltransferase 5 (Prmt5) Localizes to Chromatin Loop Anchors and Modulates Expression of Genes at TAD Boundaries During Early Adipogenesis.” eLife 12:RP88599. 10.7554/eLife.88599.1

[47] Andrews, Simon 2010. FastQC: A Quality Control Tool for High Throughput Sequence Data. Available online at: http://www.bioinformatics.babraham.ac.uk/projects/fastqc

[48] Felix, Krueger , James Frankie , Ewels Phil , Afyounian Ebrahim , Weinstein Michael , Schuster‐Boeckler Benjamin , Hulselmans Gert , and Sclamons . 2023. FelixKrueger/TrimGalore: v0.6.10 ‐ Add Default Decompression Path (0.6.10). *Zenodo*. 10.5281/zenodo.7598955

[49] Dobin, Alexander , Carrie A. Davis , Felix Schlesinger , Jorg Drenkow , Chris Zaleski , Sonali Jha , Philippe Batut , Mark Chaisson , and Thomas R. Gingeras . 2013. “STAR: Ultrafast Universal RNA‐seq Aligner.” Bioinformatics 29: 15–21. 10.1093/bioinformatics/bts635 PMC3530905 23104886

[50] Breese, Marcus R. , and Yunlong Liu . 2013. “NGSUtils: A Software Suite for Analyzing and Manipulating Next‐Generation Sequencing Datasets.” Bioinformatics 29: 494–496. 10.1093/bioinformatics/bts731 PMC3570212 23314324

[51] Liao, Yang , Gordon K. Smyth , and Wei Shi . 2014. “FeatureCounts: An Efficient General Purpose Program for Assigning Sequence Reads to Genomic Features.” Bioinformatics 30: 923–930. 10.1093/bioinformatics/btt656 24227677

[52] Chen, Yunshun , Lizhong Chen , Aaron T. L. Lun , Pedro L. Baldoni , and Gordon K. Smyth . 2025. “EdgeR v4: Powerful Differential Analysis of Sequencing Data With Expanded Functionality and Improved Support for Small Counts and Larger Datasets.” Nucleic Acids Research 53: gkaf018. 10.1093/nar/gkaf018 PMC11754124 39844453

[53] Chen, Xiyue , Christina R. Ferreira , and Shihuan Kuang . 2023. “Targeted Lipidomics Analysis of Adipose and Skeletal Muscle Tissues by Multiple Reaction Monitoring Profiling.” In Skeletal Muscle Stem Cells: Methods and Protocols (pp. 351–368). Springer US. 10.1007/978-1-0716-3036-5_25 PMC10726714 36995607

[54] Dipali, Shweta S. , Christina R. Ferreira , Luhan T. Zhou , Michele T. Pritchard , and Francesca E. Duncan . 2019. “Histologic Analysis and Lipid Profiling Reveal Reproductive Age‐Associated Changes in Peri‐Ovarian Adipose Tissue.” Reproductive Biology and Endocrinology 17: 46. 10.1186/s12958-019-0487-6 PMC6563378 31189477

[55] Xie, Zhuoer , Christina R. Ferreira , Alessandra A. Virequ , and R. Graham Cooks . 2021. “Multiple Reaction Monitoring Profiling (MRM Profiling): Small Molecule Exploratory Analysis Guided by Chemical Functionality.” Chemistry and Physics of Lipids 235: 105048. 10.1016/j.chemphyslip.2021.105048 33561466

