# Supplementary material for: Multiomics analyses reveal that PRMT5 regulates membrane transport and cholesterol synthesis in white adipocytes
Source: IMetaOmics. Author manuscript; Available in PMC 2026 Feb 5. (PMC12806136; doi:10.1002/imo2.70055)
Supplement: Supplementary Fig S1-3 — Figure S1. Prmt5AKO induces gene programs involved in metabolic and transport pathways. Figure S2. Prmt5AKO induces changes in lipid dynamics. Figure S3. Prmt5AKO alters membrane morphology in eWAT. [file NIHMS2139874-supplement-Supplementary_Fig_S1-3.pdf]

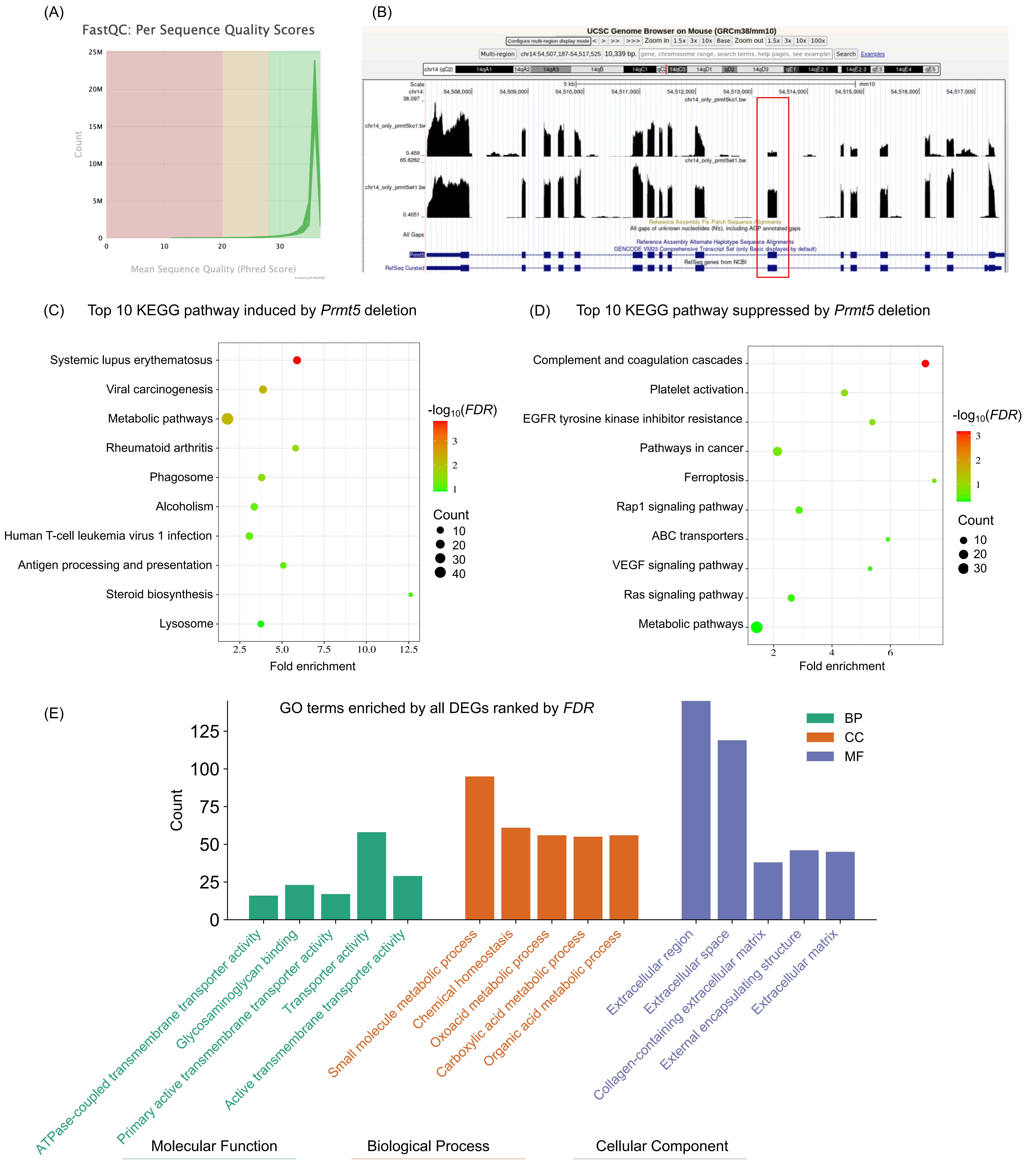
(A)
FastQC: Per Sequence Quality Scores
Count
25M
20M
15M
10M
5M
0
0
10
20
30
Mean Sequence Quality (Phred Score)
(B)
UCSC Genome Browser on Mouse (GRCm38/mm10)
chr14:54,507,187-54,517,525
10,339 bp.
chr14_only_prmt5ko1.bw
chr14_only_prmt5wt1.bw
All Gaps
Prmt5
RefSeq Curated
RefSeq genes from NCBI
(C)
Top 10 KEGG pathway induced by *Prmt5* deletion
Systemic lupus erythematosus
Viral carcinogenesis
Metabolic pathways
Rheumatoid arthritis
Phagosome
Alcoholism
Human T-cell leukemia virus 1 infection
Antigen processing and presentation
Steroid biosynthesis
Lysosome
2.5
5.0
7.5
10.0
12.5
Fold enrichment
-log10(FDR)
3
2
1
Count
10
20
30
40
(D)
Top 10 KEGG pathway suppressed by *Prmt5* deletion
Complement and coagulation cascades
Platelet activation
EGFR tyrosine kinase inhibitor resistance
Pathways in cancer
Ferroptosis
Rap1 signaling pathway
ABC transporters
VEGF signaling pathway
Ras signaling pathway
Metabolic pathways
2
4
6
Fold enrichment
-log10(FDR)
3
2
1
Count
10
20
30
(E)
GO terms enriched by all DEGs ranked by *FDR*
Count
125
100
75
50
25
0
BP
CC
MF
ATPase-coupled transmembrane transporter activity
Glycosaminoglycan binding
Primary active transmembrane transporter activity
Transporter activity
Active transmembrane transporter activity
Small molecule metabolic process
Chemical homeostasis
Oxoacid metabolic process
Carboxylic acid metabolic process
Organic acid metabolic process
Extracellular region
Extracellular space
Collagen-containing extracellular matrix
External encapsulating structure
Extracellular matrix
Molecular Function
Biological Process
Cellular Component

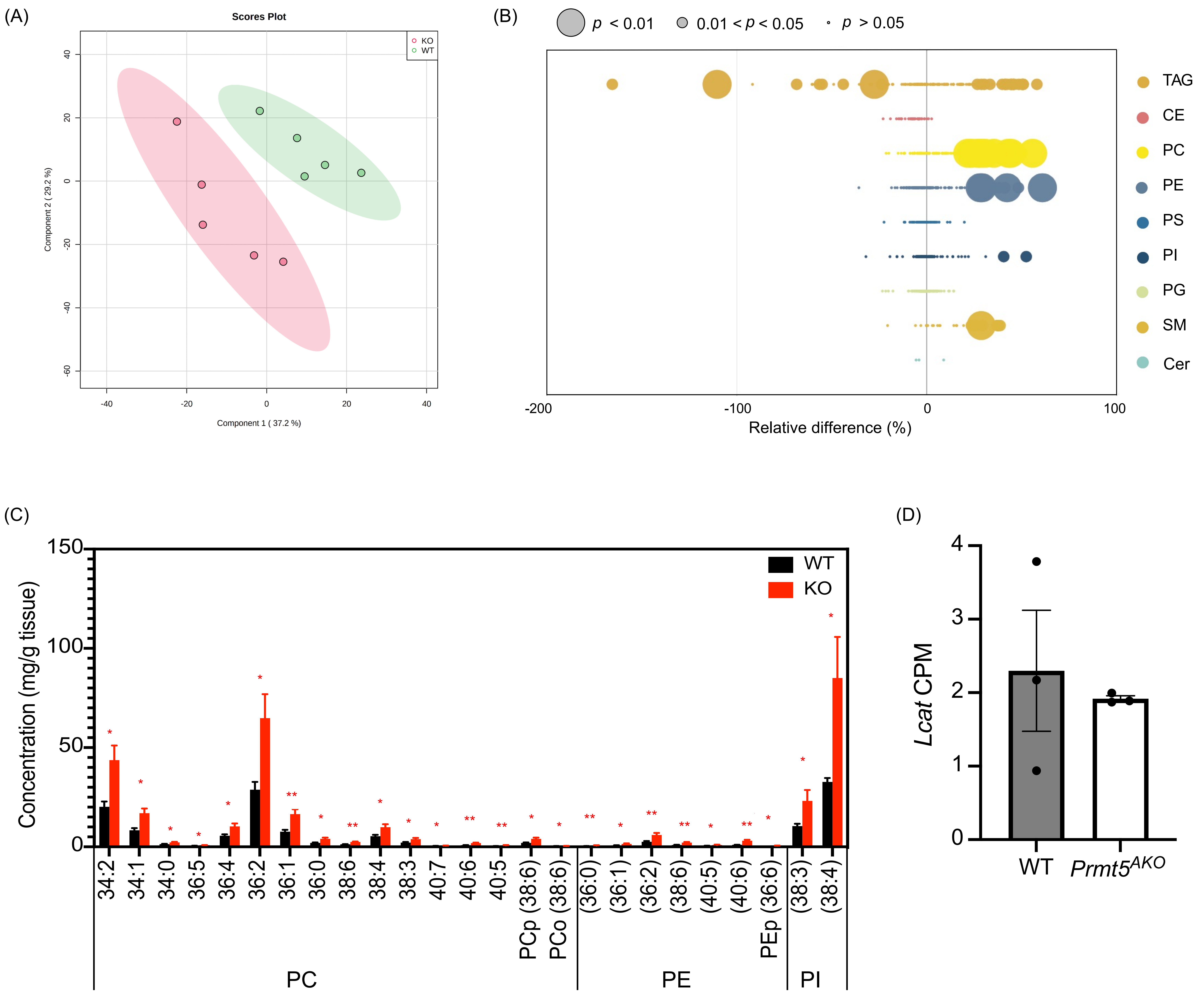
(A)
Scores Plot
KO
WT
Component 1 (37.2 %)
Component 2 (29.2 %)
(B)
p < 0.01
0.01 < p < 0.05
p > 0.05
TAG
CE
PC
PE
PS
PI
PG
SM
Cer
Relative difference (%)
(C)
Concentration (mg/g tissue)
WT
KO
34:2
34:1
34:0
36:5
36:4
36:2
36:1
36:0
38:6
38:4
38:3
40:7
40:6
40:5
PCp (38:6)
PCo (38:6)
(36:0)
(36:1)
(36:2)
(38:6)
(40:5)
(40:6)
PEp (36:6)
(38:3)
(38:4)
PC
PE
PI
(D)
Lcat CPM
WT
Prmt5AKO

(A)

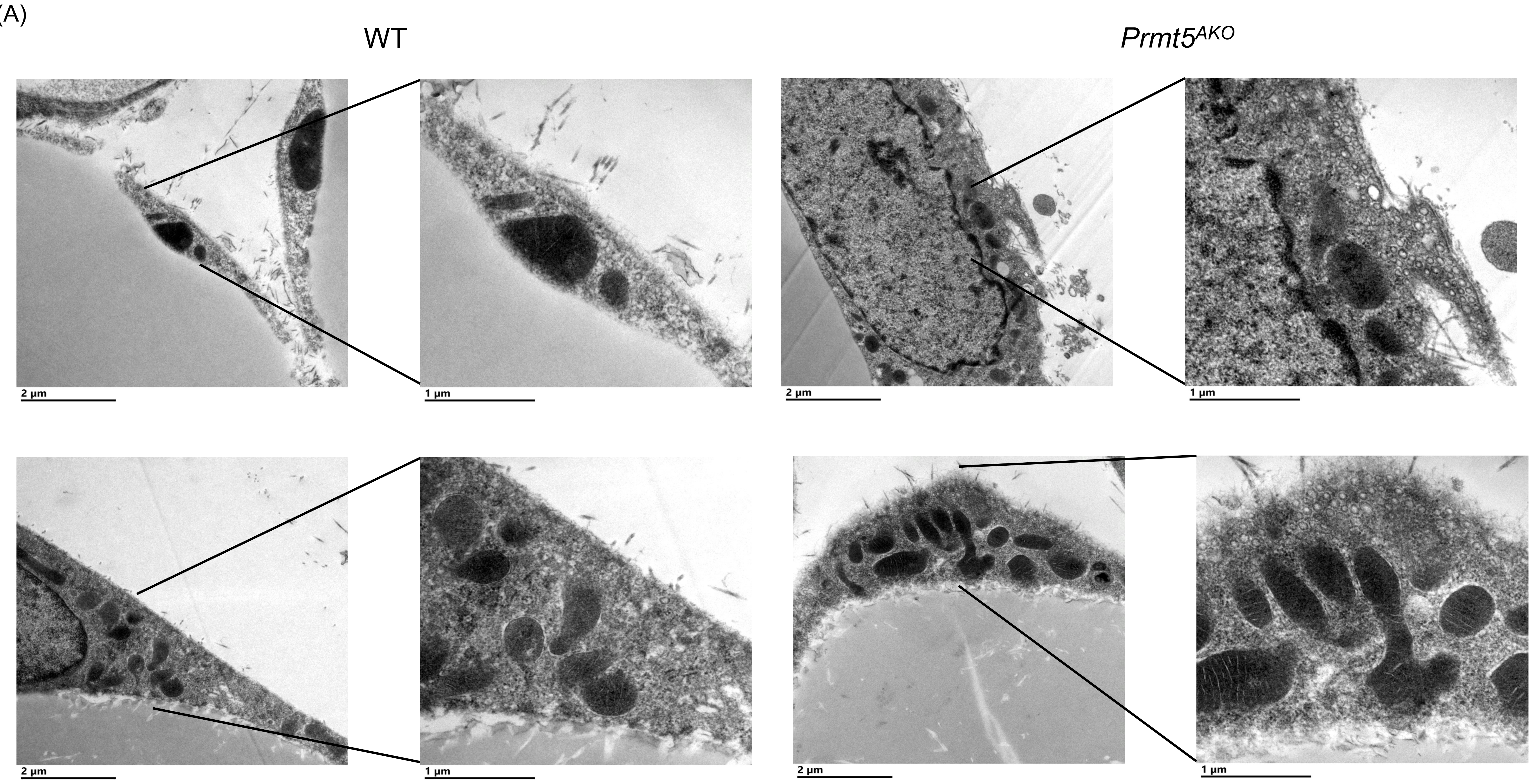
WT
Prmt5^AKO
2 µm
1 µm
2 µm
1 µm
2 µm
1 µm
2 µm
1 µm

(B)

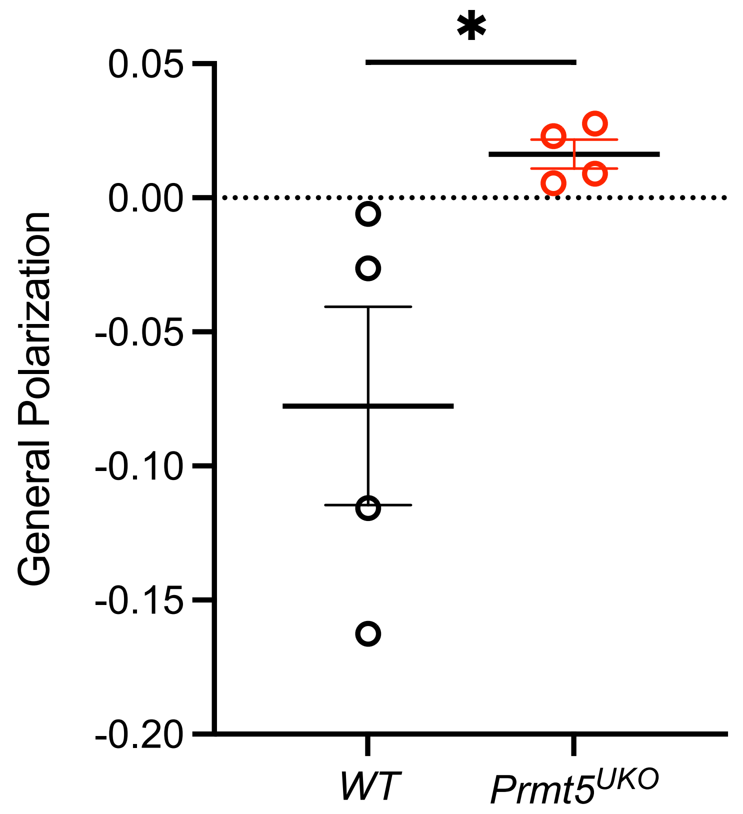
*
0.05
0.00
-0.05
-0.10
-0.15
-0.20
General Polarization
WT
Prmt5^UKO
